# Supplementary material for: Rapid cochlear gene therapy in adult deaf mice: Vglut3 rescue via AAV8 achieves day-1 hearing restoration
Source: Mol Ther Methods Clin Dev. 2025 Jul 21;33(3):101539. doi: 10.1016/j.omtm.2025.101539 (PMC12337667; doi:10.1016/j.omtm.2025.101539)
Supplement: Document S1. Figures S1–S7 [file mmc1.pdf]

OMTM, Volume *33*

Supplemental information

Rapid cochlear gene therapy in adult deaf mice: *Vglut3* rescue via AAV8 achieves day-1 hearing restoration

Ting Zhang, Rongqun Zhai, Mengli Liu, Hongen Xu, Liang Wang, Wenxue Tang, Bei Chen, and Xingle Zhao

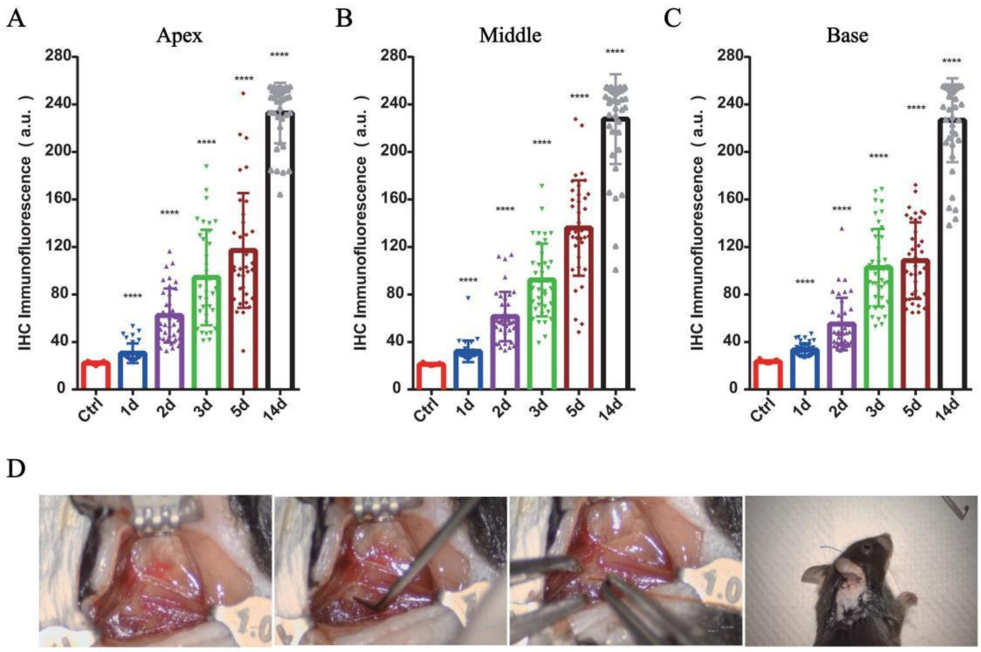

Figure S1. Quantification of GFP Fluorescence Intensity in the Cochlea Following AAV8-GFP Injection.

(A–C) Quantification of GFP fluorescence intensity in the apical (A), middle (B), and basal (C) turns of the cochlea at different time points post-injection (1, 2, 3, 5, and 14 days). Data are presented as mean ± SD. ****$p < 0.0001$. (D) Images of critical step of the adult posterior semicircular canal (PSCC) surgical approach.

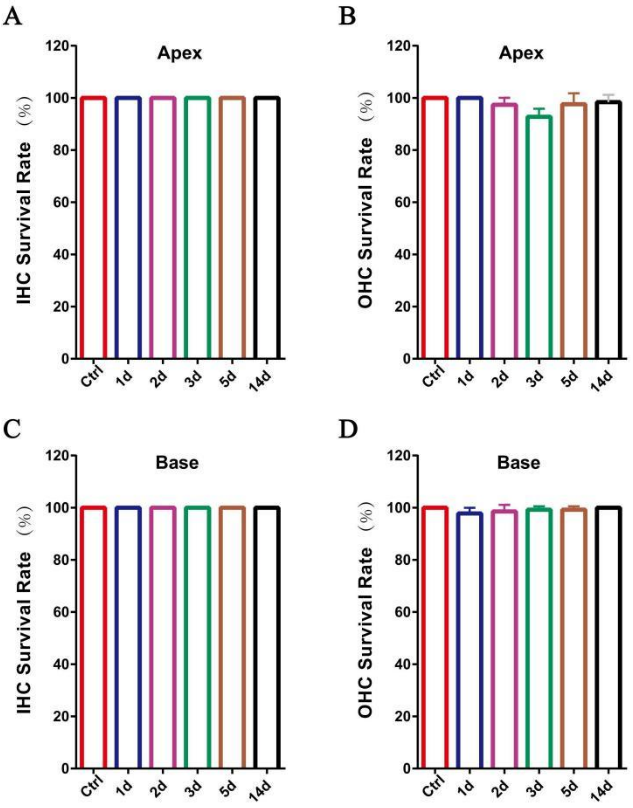

Figure S2. Hair Cell Survival in the Apical and Basal Turns of the Cochlea Following AAV8-GFP Injection

(A-D) Quantification of IHC and OHC survival rates in the apical (A, B) and basal (C, D) turns at various post-injection time points.Data are presented as mean ± SEM (N = 3, one-way ANOVA, $p > 0.05$)..

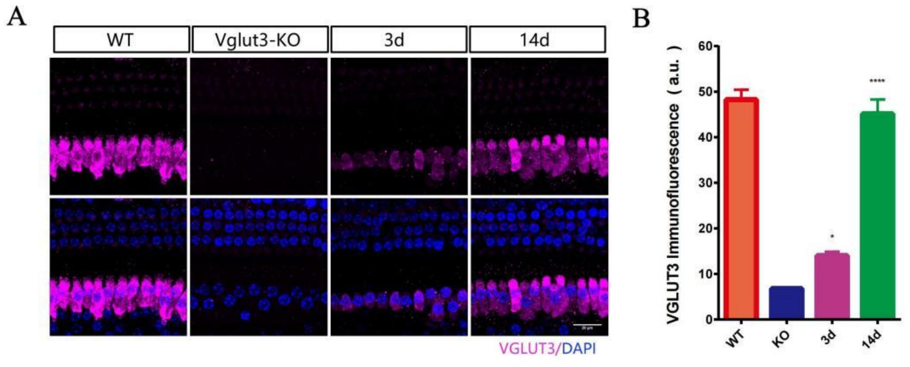

Figure S3. Temporal Dynamics of VGLUT3 Expression in the Adult Mouse Cochlea Following AAV8-*Vglut3*-FLAG Gene Therapy.

(A) Representative immunofluorescence images showing VGLUT3 expression in the middle turn of the cochlea at 3 and 14 days post-injection. Scale bar: 20 µm. (B) Quantification of VGLUT3 fluorescence intensity in the middle cochlear turn at different post-injection time points. Data are presented as mean ± SEM (n = 34–36 IHCs, N=3 mice per group). One-way ANOVA, $*p < 0.05$, $****p < 0.0001$.

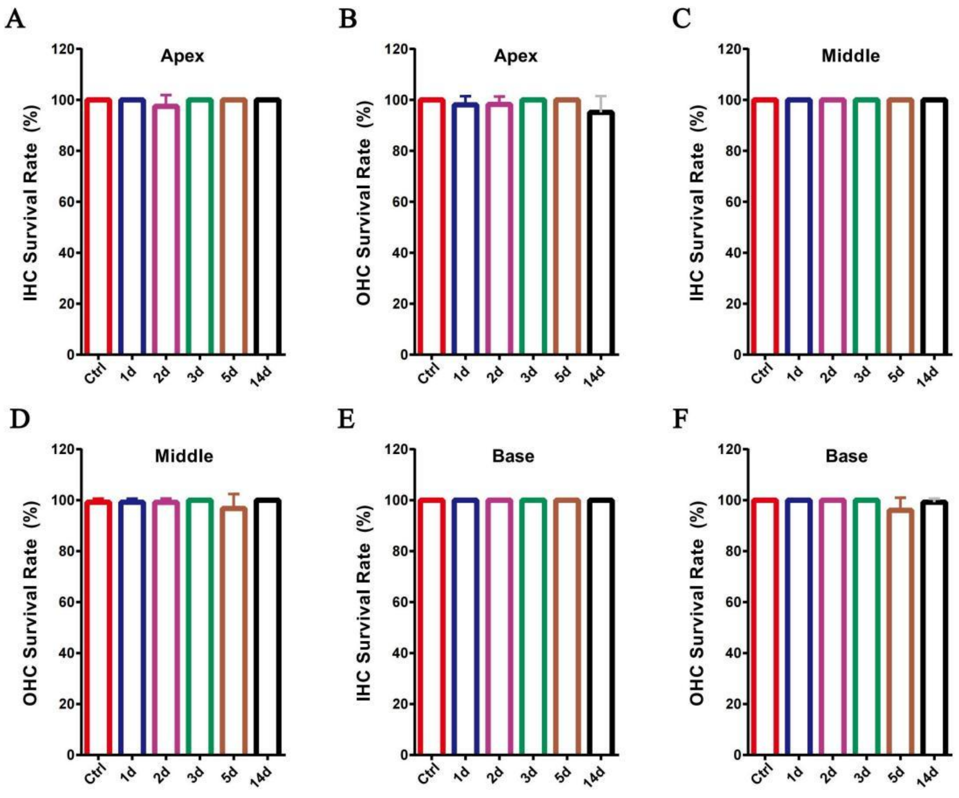

Figure S4. Hair cell survival in the apical, middle, and basal turns of the cochlea following AAV8-*Vglut3*-FLAG injection.

(A-F) Quantification of IHC and OHC survival rates in the apical (A, B), middle (C, D), and basal (E, F) cochlear turns at 1, 2, 3, 5, and 14 days post-injection. Data are presented as mean ± SEM (N = 3, one-way ANOVA, $p > 0.05$)..

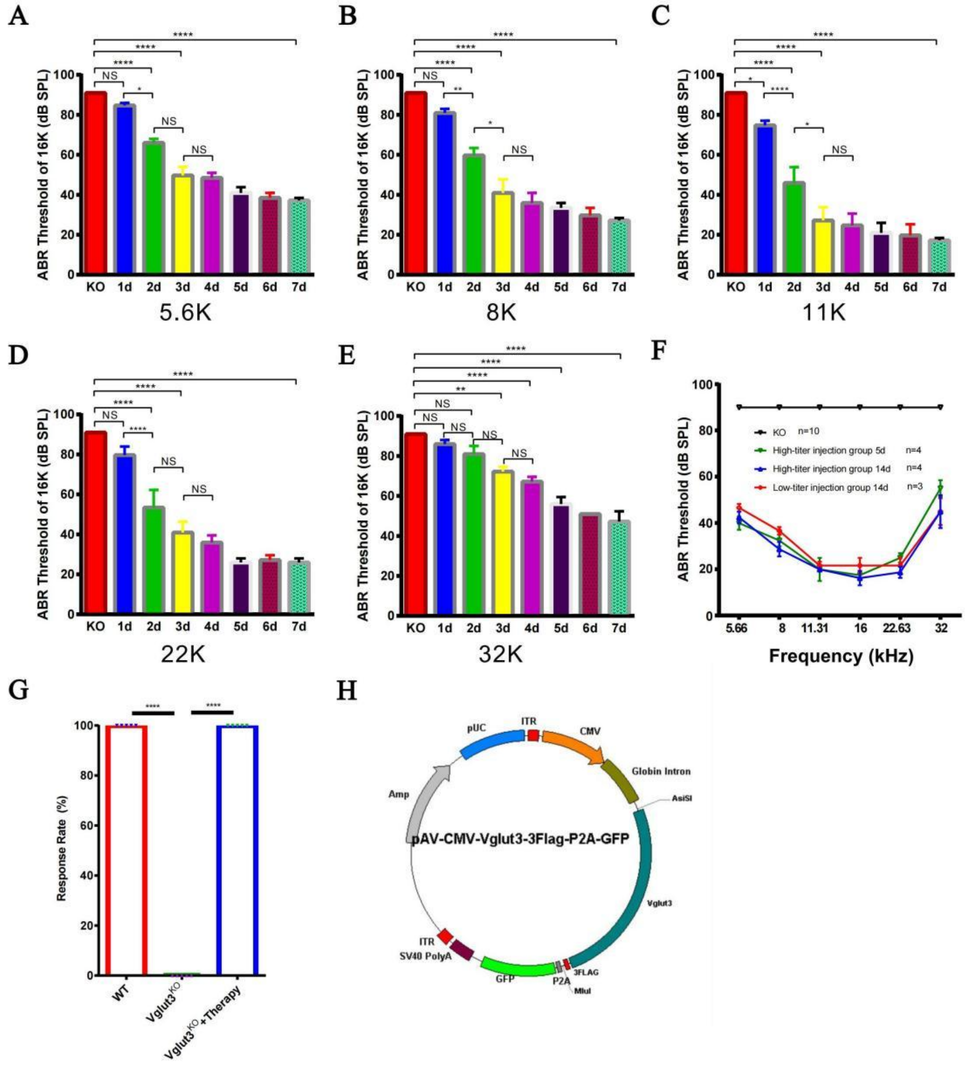

Figure S5. ABR thresholds Changes Following AAV8-*Vglut3*-FLAG Gene Therapy.

(A-E) Comparison of ABR thresholds at 5.66(A), 8(B), 11.31(C), 22.63(D), 32 (E)kHz between untreated *Vglut3*^{KO} mice and treated *Vglut3*^{KO} at different post-injection time points. (F) Quantification of ABR thresholds in *Vglut3*^{KO} Mice with different Viral Titers. (G) Ear movement response rates to 90 dB stimuli in WT, *Vglut3*^{KO}, and *Vglut3*^{KO} mice after 2 weeks of treatment. (H) Schematic representation of the vector.

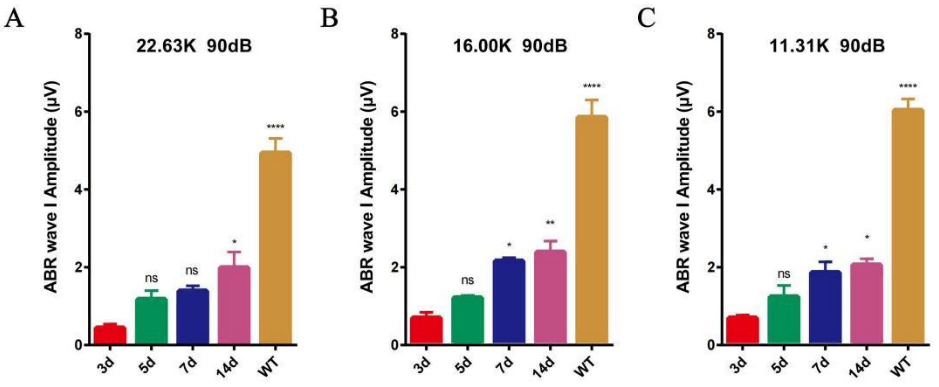

Figure S6. ABR Wave I Amplitude Changes Following AAV8-*Vglut3*-FLAG Gene Therapy.

(A-C) Quantification of ABR wave I amplitudes at 11.31 kHz (A), 16 kHz (B), and 22.63 kHz (C) at a sound pressure level of 90 dB across different post-injection time points. Data are presented as mean ± SEM.

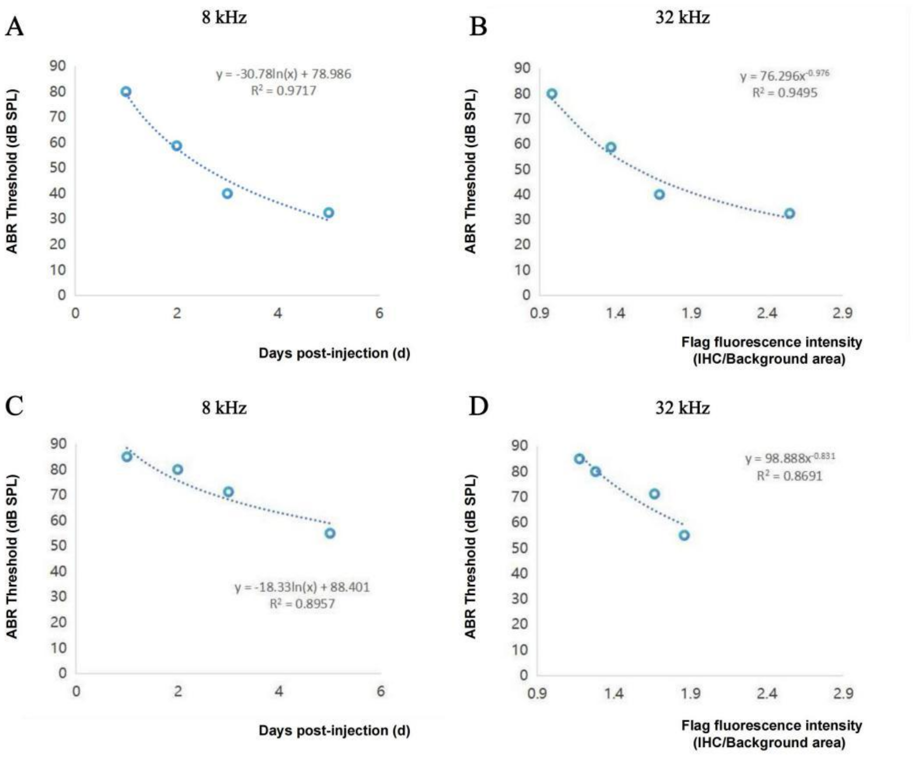

Figure S7. Quantitative Analysis of ABR Threshold Recovery and Its Correlation with *Vglut3* Expression.

(A) Logarithmic regression curves depicting the time-dependent decline in ABR thresholds at 8 kHz following gene therapy. (B) Power-law regression curves

illustrating the inverse relationship between FLAG fluorescence intensity (surrogate for *Vglut3* expression) and ABR thresholds at 8 kHz. (C) Logarithmic regression curves depicting the time-dependent decline in ABR thresholds at 32 kHz following gene therapy. (D) Power-law regression curves illustrating the inverse relationship between FLAG fluorescence intensity (surrogate for *Vglut3* expression) and ABR thresholds at 32 kHz.
