# Supplementary material for: Rapid cochlear gene therapy in adult deaf mice: Vglut3 rescue via AAV8 achieves day-1 hearing restoration
Source: Mol Ther Methods Clin Dev. 2025 Jul 21;33(3):101539. doi: 10.1016/j.omtm.2025.101539 (PMC12337667; doi:10.1016/j.omtm.2025.101539)
Supplement: Document S2. Article plus supplemental information [file mmc2.pdf]

Molecular Therapy
Methods & Clinical Development
Original Article

Rapid cochlear gene therapy in adult deaf mice: *Vglut3* rescue via AAV8 achieves day-1 hearing restoration

Ting Zhang,[1,4] Rongqun Zhai,[1,4] Mengli Liu,[2,4] Hongen Xu,[2] Liang Wang,[1] Wenxue Tang,[3] Bei Chen,[1] and Xingle Zhao[1]

[1]Department of Otolaryngology Head and Neck Surgery, The First Affiliated Hospital of Zhengzhou University, Zhengzhou 450000, China; [2]Precision Medicine Center, Academy of Medical Sciences, Zhengzhou University, Zhengzhou 450052, China; [3]The Research and Application Center of Precision Medicine, The Second Affiliated Hospital of Zhengzhou University, Zhengzhou 450014, China

Genetic hearing loss, caused by mutations in critical auditory genes, has seen promising advances through gene therapy, yet the temporal dynamics of early-stage auditory functional recovery and therapeutic transgene expression patterns following intervention remain uncharacterized in preclinical deafness models. This study systematically investigates the post-treatment progression of cochlear functional restoration and spatially resolved transgene expression kinetics in adult *Vglut3* knockout (*Vglut3^{KO}*) mice following adeno-associated virus (AAV)-mediated inner ear gene therapy. AAV8 vectors delivering *Vglut3* were injected via the posterior semicircular canal (PSCC), with auditory brainstem response (ABR) thresholds and cochlear transgene expression assessed at days 1–14 post-injection. VGLUT3 expression in *Vglut3^{KO}* mice revealed rapid transduction, detectable in inner hair cells (IHCs) by day 1, peaking at day 14. Remarkably, hearing recovery commenced as early as day 1 post-injection, and plateaued near wild-type (WT) levels by day 5. Functional correlation analysis demonstrated a robust inverse relationship between FLAG expression and hearing thresholds. This study provides critical insights into the dynamic processes underlying cochlear gene therapy and challenges the conventional paradigm that 1–2 weeks are required for functional recovery.

INTRODUCTION

Hearing loss represents one of the most prevalent sensory disorders worldwide, affecting over 466 million individuals, with genetic mutations accounting for approximately 50%–60% of congenital cases.[1] Among hereditary forms of deafness, defects in synaptic transmission between inner hair cells (IHCs) and spiral ganglion neurons (SGNs) are increasingly recognized as critical contributors to auditory dysfunction.[2,3] The vesicular glutamate transporter 3 (*Vglut3/Slc17a8*) gene, which encodes the protein responsible for packaging glutamate into synaptic vesicles of IHCs, has emerged as a pivotal player in this process.[3] Mutations in *Vglut3* disrupt glutamate release at the IHC-SGN synapse, leading to profound congenital deafness in both humans and mouse models.[3] While cochlear implants partially restore hearing in some cases, they fail to address the underlying biological deficits, highlighting the urgent need for therapies that directly target the molecular basis of synaptic dysfunction.[4]

Recent advances in adeno-associated virus (AAV)-mediated gene therapy have revolutionized the treatment of monogenic disorders, with the inner ear presenting a uniquely accessible target for localized intervention.[4–12] The cochlea's isolated fluid compartments and immune-privileged status enable efficient AAV delivery while minimizing systemic exposure.[5] Preclinical studies in murine models of deafness—including *Otof$^{-/-}$*, *Tmc1$^{-/-}$*, and *Tmprss3* mutants—have demonstrated the potential of AAV vectors to restore auditory function by rescuing defective genes.[13–16] However, prior studies have predominantly assessed outcomes at weekly or monthly intervals,[13,16,17] leaving the early post-injection period—when therapeutic effects may first manifest—poorly characterized. Critical gaps persist in our understanding of the temporal dynamics governing transgene expression and functional recovery following inner ear gene therapy. Furthermore, the relationship between the kinetics of transgene expression and hearing restoration remains undefined, limiting our ability to optimize dosing regimens and predict therapeutic windows.

The choice of AAV serotype and delivery route profoundly impact transduction efficiency and safety.[11,18–23] AAV8, noted for its

Received 26 March 2025; accepted 16 July 2025;
https://doi.org/10.1016/j.omtm.2025.101539.

[4]These authors contributed equally

Correspondence: Wenxue Tang, The Research and Application Center of Precision Medicine, The Second Affiliated Hospital of Zhengzhou University, Zhengzhou 450014, China.
E-mail: twx@zzu.edu.cn
Correspondence: Bei Chen, Department of Otolaryngology Head and Neck Surgery, The First Affiliated Hospital of Zhengzhou University, Zhengzhou, China.
E-mail: chenbei815@163.com
Correspondence: Xingle Zhao, Department of Otolaryngology Head and Neck Surgery, The First Affiliated Hospital of Zhengzhou University, Zhengzhou, China.
E-mail: 2308985070@qq.com

© 2025 The Author(s). Published by Elsevier Inc. on behalf of The American Society of Gene and Cell Therapy.
This is an open access article under the CC BY license (http://creativecommons.org/licenses/by/4.0/).

tropism toward hair cells and neuronal tissues, has shown promise in cochlear gene delivery.[24] Yet, conventional injection methods such as cochleostomy or round window membrane penetration risk mechanical trauma, inflammation, and transient threshold shifts.[25,26] Emerging approaches, including posterior semicircular canal (PSCC) delivery, leverage the interconnected endolymphatic system to distribute viral vectors throughout the cochlea and vestibular organs while minimizing surgical damage.[27] Despite these advances, the safety profile of AAV8 in the mature murine inner ear—particularly its acute effects on hair cell viability and auditory thresholds—remains underexplored.

A key barrier of precision gene therapy for the inner ear lies in the paucity of high-resolution temporal data linking molecular rescue to functional recovery. Our previous work in *Vglut3*^{KO} mice revealed that AAV-mediated *Vglut3* delivery restores auditory brainstem response (ABR) thresholds within 1–2 weeks.[27] However, these studies did not address when transgene expression initiates, how rapidly synaptic transmission recovers, or whether early molecular changes correlate with measurable hearing improvements. Such knowledge is essential for refining therapeutic strategies, particularly for congenital deafness, where delayed intervention may compromise neural plasticity.

This study bridges these gaps by establishing the first day-by-day timeline of AAV8-mediated gene expression and hearing recovery in the adult murine cochlea. Using dual approaches—AAV8-GFP to map transduction kinetics and AAV8-*Vglut3*-FLAG to assess therapeutic efficacy—we systematically evaluated the following: (1) the spatiotemporal dynamics of auditory recovery and transgene expression in inner ear; (2) the safety profile of posterior semicircular canal-delivered AAV in the acute phase; and (3) the functional relationship between VGLUT3 expression levels and ABR threshold recovery. Our findings challenge prevailing assumptions about the latency of therapeutic effects in mature auditory systems, revealing hearing improvements as early as 24 h post-injection.

RESULTS

Spatiotemporal dynamics of AAV8-GFP expression in the mouse cochlea post-injection

To investigate the early temporal changes in cochlear gene expression following inner ear gene therapy, we first examined the spatiotemporal expression of GFP (green fluorescent protein) after AAV8-GFP injection. AAV8-GFP was delivered into the cochlea of 4-week-old wild-type (WT) mice via PSCC (Figure S1D). Mice were sacrificed at 1, 2, 3, 5, 9, and 14 days post-injection; control groups consisted of age-matched (4-week-old) WT mice without injection. The cochleae were harvested, decalcified, and whole-mounted for immunofluorescence staining. GFP expression was analyzed across the apical, middle, and basal turns of the cochlea. The results showed that GFP fluorescence was first detected at day 1 post-injection, with weak but distinct expression in several IHCs of the cochlea (Figures 1A, 1C, and 1E). By day 3, GFP signal intensity had markedly increased and extended to most IHCs throughout the cochlea, while by day 5, robust GFP expression was observed throughout the entire cochlea, including the apical, middle, and basal turns. The fluorescence signal remained significant increase through day 14 (Figures 1A, 1C, and 1E). However, we did not observe any detectable GFP expression in OHCs following viral injection, consistent with our previous studies using AAV8 vectors.[27] While some reports have demonstrated AAV8-mediated OHC transduction,[20] these discrepancies likely stem from variations in viral production protocols (including purification methods and titer quantification), experimental conditions (such as injection technique and volume), or developmental stages at the time of injection.

To quantify GFP expression over time, we used ImageJ software to measure fluorescence intensity at different post-injection time points (Figure S1). The analysis revealed a significant increase in GFP fluorescence in the apical (30.6 ± 8.2 vs. 22.2 ± 1.1, n = 34 and 35 IHCs, N = 3 mice per groups, one-way ANOVA, $p < 0.0001$), middle (32.3 ± 9.1 vs. 21.3 ± 0.8, n = 33 and 37 IHCs, N = 3 mice per groups, one-way ANOVA, $p < 0.0001$), and basal (32.5 ± 5.6 vs. 22.6 ± 1.1, n = 38 and 37 IHCs, N = 3 mice per groups, one-way ANOVA, $p < 0.0001$) turns of the cochleae as early as day 1 post-injection, indicating rapid initiation of transgene expression. Fluorescence intensity continued to rise progressively over time, reaching a peak at day 14, with an approximately 6-fold increase compared to the initial levels at day 1.

To eliminate the influence of background fluorescence, we normalized the measured fluorescence intensity of IHCs by dividing it by the fluorescence intensity of the surrounding background area (Figures 1B, 1D, and 1F). The analysis confirmed a continuous increase in GFP fluorescence intensity within IHCs following AAV8-GFP injection. By day 3 post-injection, the fluorescence intensity in the apical (2.10 ± 0.77 vs. 1.00 ± 0.05, n = 35 and 35 IHCs, N = 3 mice per groups, one-way ANOVA, $p < 0.01$), middle (2.27 ± 0.80 vs. 1.00 ± 0.04, n = 37 and 37 IHCs, N = 3 mice per groups, one-way ANOVA, $p < 0.001$), and basal (2.97 ± 1.06 vs. 1.00 ± 0.05, n = 40 and 37 IHCs, N = 3 mice per groups, one-way ANOVA, $p < 0.0001$) turns of the cochleae exhibited a statistically significant increase compared to control group, indicating robust and progressive transgene expression in cochlear hair cells.

Spatiotemporal dynamics of AAV8-GFP expression in the utricle of adult mice post-injection

Since the vestibular organs and the cochlea are interconnected through endolymphatic fluid, AAV8-GFP delivered via PSCC injection also transduced vestibular organs. To investigate the temporal dynamics of AAV8-GFP expression in the vestibular organs, utricles were harvested at 1, 2, 3, 5, and 14 days post-injection, followed by immunofluorescence staining to assess GFP expression in different regions. The results showed a detectable increase in GFP fluorescence intensity as early as day 1 post-injection (Figure 2). The fluorescence signal continued to intensify over time, reaching its peak at day 14, suggesting a progressive and stable expression of the transgene in the utricle (Figure 2).

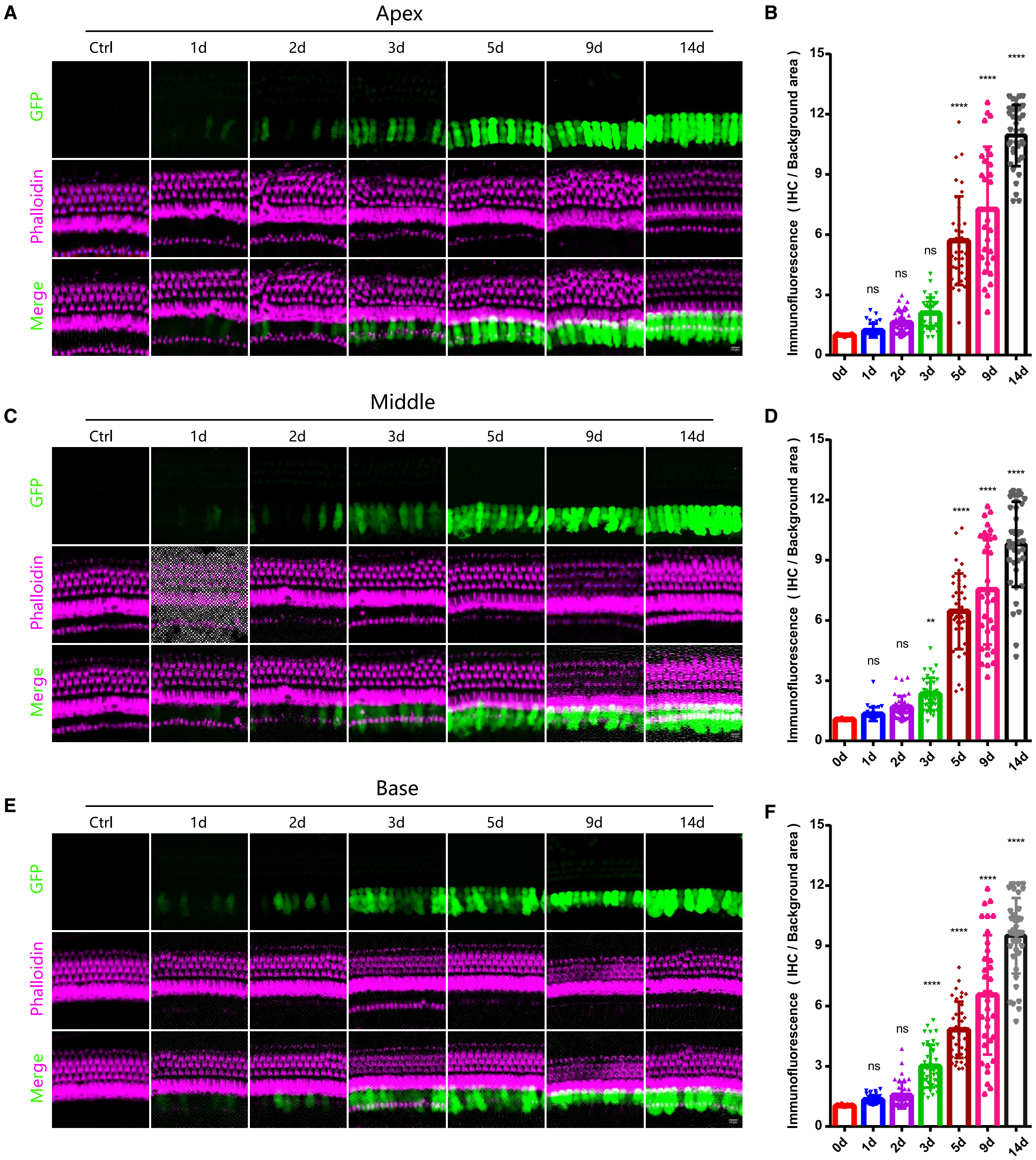

Figure 1. Temporal dynamics of AAV8-GFP expression in the cochlea of adult mice

(A, C, and E) Representative immunofluorescence images of GFP expression in the cochlea of wild-type mice at 1, 2, 3, 5, 9, and 14 days post-AAV8-GFP injection. Images are shown for the apical, middle, and basal turns of the cochlea. Scale bars: 10 μm. (B, D, and F) Quantification of GFP fluorescence intensity in the apical, middle, and basal turns of the cochlea at different time points post-injection. Data are presented as mean ± SD. $^{**}p < 0.01$, $^{***}p < 0.001$, and $^{****}p < 0.0001$.

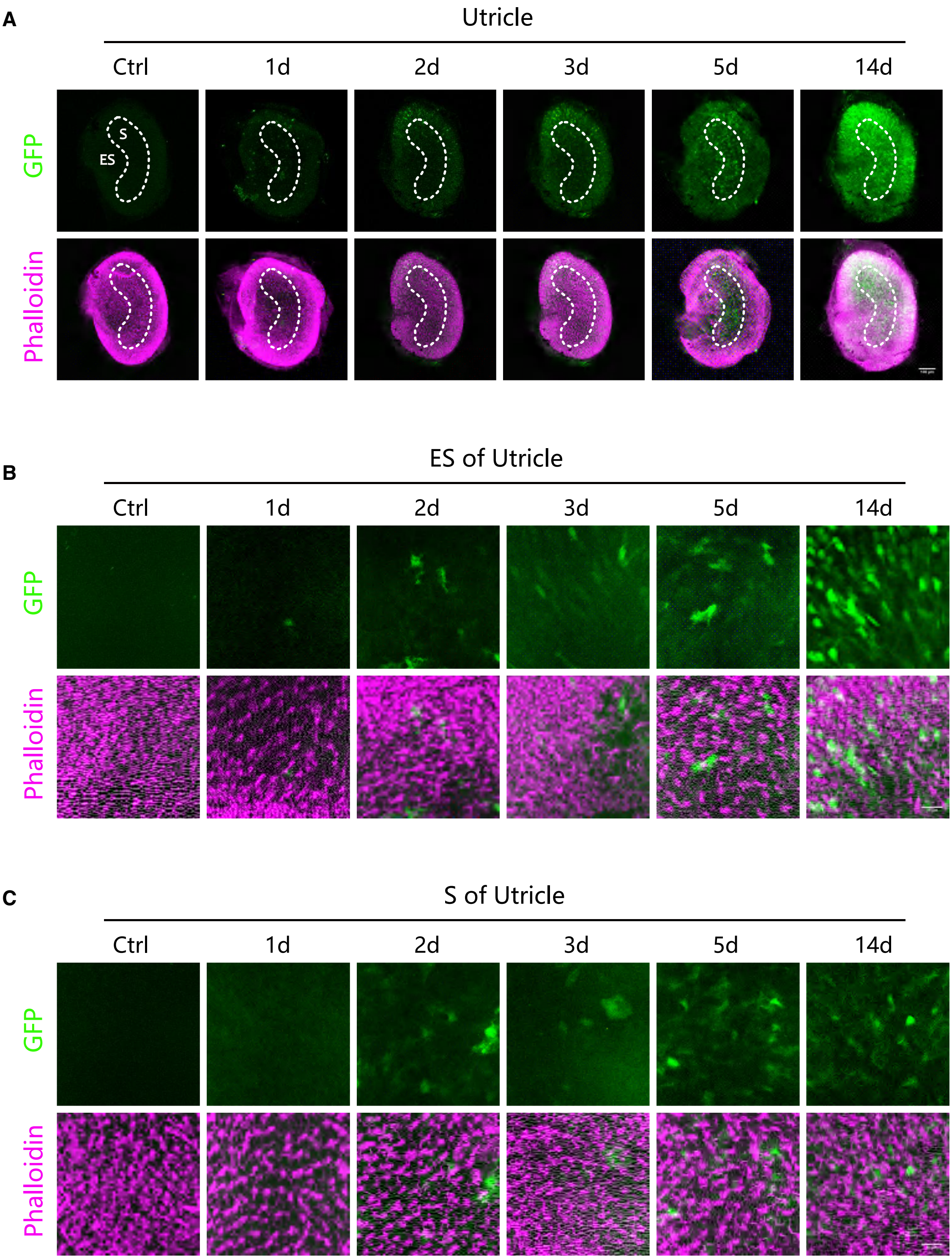

Figure 2. Temporal dynamics of AAV8-GFP expression in the utricle following posterior semicircular canal injection
(A) Representative immunofluorescence images showing GFP expression in the utricle at different time points post-injection (0, 1, 2, 3, 5, and 14 days). Scale bars: 100 μm. (B) Higher magnification images highlighting GFP expression in the ES region at day 0, 1, 2, 3, 5, and 14 post-injection. Scale bars: 20 μm. (C) Higher magnification images highlighting GFP expression in the S region at day 0, 1, 2, 3, 5, and 14 post-injection. Scale bars: 20 μm.

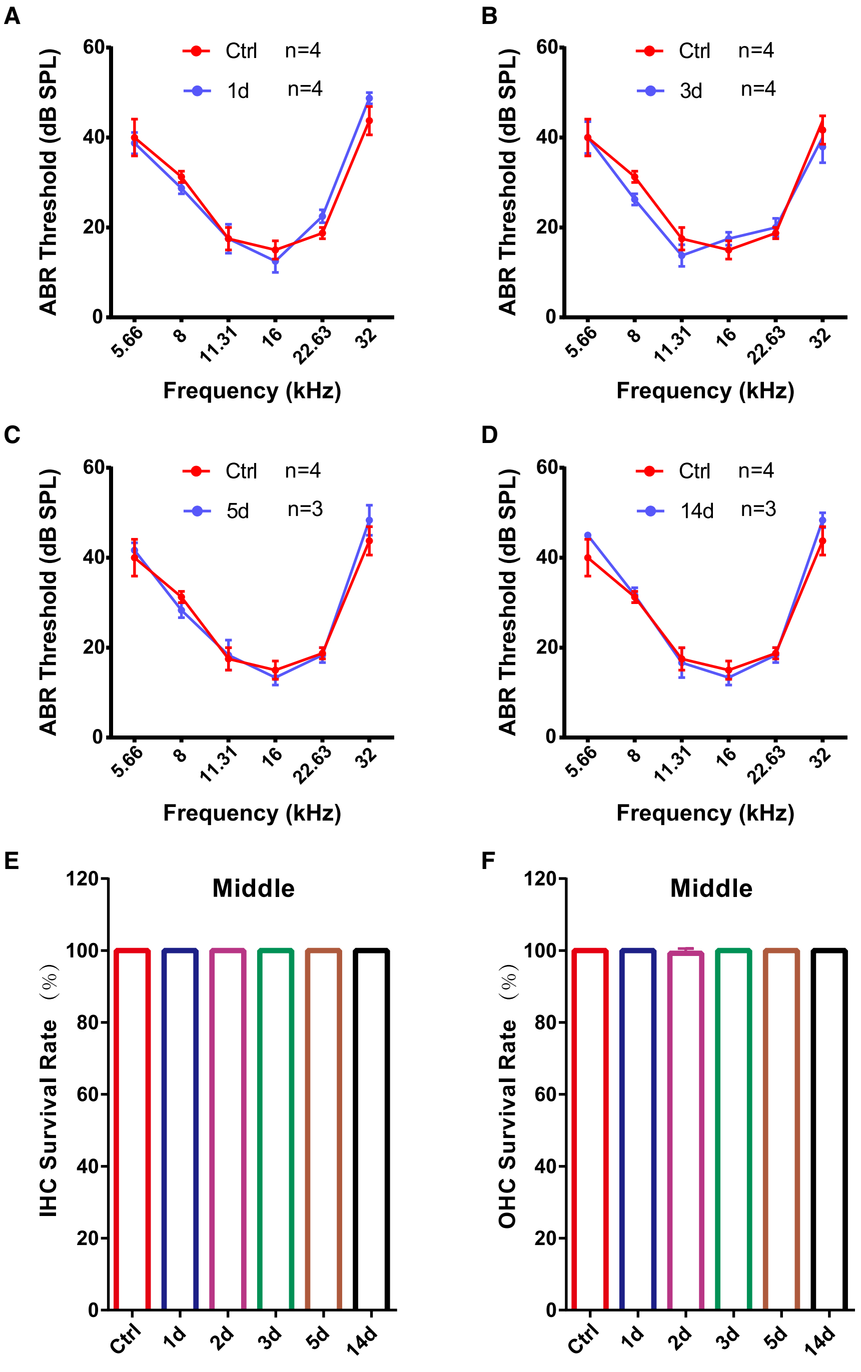

Figure 3. Safety assessment of AAV8-GFP injection in the inner ear of adult WT mice

(A–D) Auditory brainstem response (ABR) thresholds at 5.66, 8, 11.31, 16, 22.63, and 32 kHz were measured in AAV8-GFP-injected mice at 1 (A), 3 (B), 5 (C), and 14 (D) days post-injection and compared to untreated control mice. Data are presented as mean ± SEM. Two-way ANOVA, $p > 0.05$. (E and F) Quantification of IHC (E) and OHC (F) survival rates in the middle turn at various post-injection time points. Statistical analysis confirmed no significant difference in hair cell survival between AAV8-GFP-injected and control cochleae (one-way ANOVA, $p > 0.05$).

The utricle can be divided into the extrastriola (ES) region and the striola (S) region based on the distribution and function of hair cells.[28] The S region is centrally located in the utricle with a curved band-like structure, predominantly containing type I hair cells, which are involved in detecting rapid head movements.[28] The ES region, situated peripherally around the S region, mainly consists of type II hair cells, which contribute to postural stability and low-frequency stimulus perception.[28] Our study revealed that GFP expression in the ES region appeared earlier and exhibited a stronger signal intensity compared to the S region (Figure 2), suggesting differential viral transduction efficiency and cellular uptake between these two regions.

AAV8-GFP injection preserves acute-phase auditory function and hair cell integrity

To assess the acute-phase safety profile of AAV8-GFP injection in the inner ear, we measured ABR in injected mice at 1, 3, 5, and 14 days post-injection and compared them to untreated controls (Figure 3). Our results showed that ABR thresholds remained unchanged at all examined time points, including 1 day post-injection (38.8 ± 4.8, 28.8 ± 2.5, 17.5 ± 6.5, 12.5 ± 5.0, 22.5 ± 2.9, and 48.8 ± 2.5 dB vs. 40.0 ± 8.2, 31.3 ± 2.5, 17.5 ± 5.0, 15.0 ± 4.1, 18.8 ± 2.5, and 43.8 ± 6.3 dB at 5.66, 8, 11.31, 16, 22.63, and 32 kHz; $N = 4$ mice, two-way ANOVA, $p > 0.05$, Figure 3A), indicating that AAV8-GFP injection did not cause any detectable auditory impairment. These findings suggest that AAV8-GFP delivery via PSCC is well tolerated and does not compromise auditory function in adult WT mice.

To assess whether AAV8-GFP injection caused any damage to cochlear hair cells, we quantified the survival rates of IHCs and outer hair cells (OHCs) at different time points post-injection across the apical, middle, and basal turns of the cochlea. Immunofluorescence staining for phalloidin was performed on whole-mount cochlear preparations at 1, 2, 3, 5, and 14 days post-injection. The results showed that at all examined time points, both IHCs and OHCs remained intact with no significant hair cell loss in any cochlear region compared to the control group (Figures 3E, 3F, and S2). These findings indicate that AAV8-GFP administration via PSCC does not compromise hair cell viability

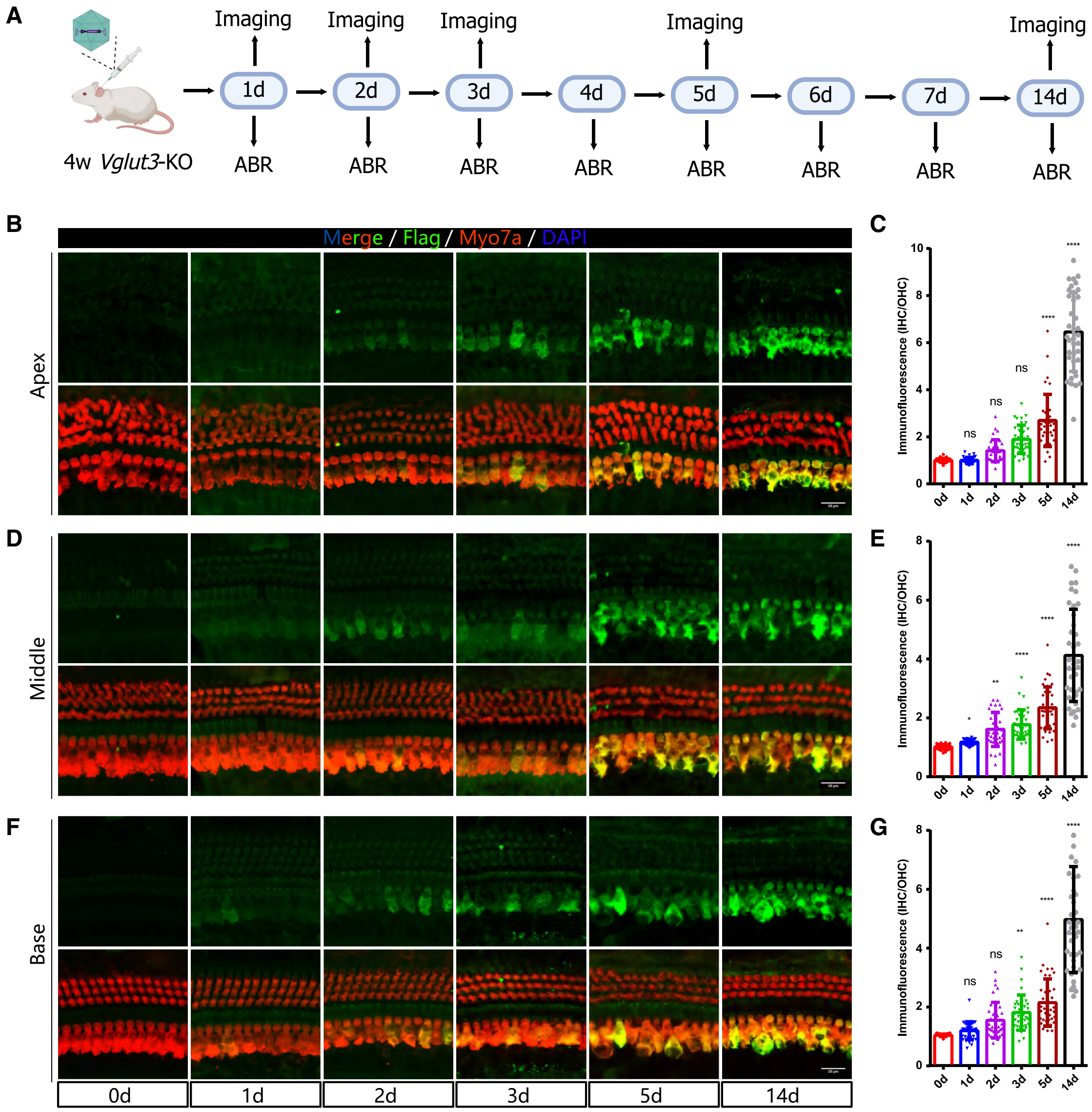

Figure 4. Temporal dynamics of FLAG expression in the adult mouse cochlea following AAV8-*Vglut3*-FLAG gene therapy

(A) Schematic representation of ABR measurements and immunofluorescence analysis at different time points following AAV8-*Vglut3*-FLAG injection in *Vglut3*^{KO} mice. (B, D, and F) Representative immunofluorescence images showing FLAG expression in the apical, middle, and basal turns of the cochlea at 1, 2, 3, 5, and 14 days post-injection. Scale bars: 20 μm. (C, E, and G) Quantification of FLAG fluorescence intensity in the apical, middle, and basal cochlear turns at different post-injection time points. Fluorescence intensity was normalized to background fluorescence to account for potential variations in imaging conditions. Data are presented as mean ± SD. One-way ANOVA, $^{*}p < 0.05$, $^{**}p < 0.01$, and $^{****}p < 0.0001$.

in the short term, supporting the safety of this gene delivery approach in the adult mouse inner ear.

Spatiotemporal dynamics of VGLUT3 expression in the mouse cochlea following gene therapy

To investigate the temporal expression pattern of the AAV-delivered gene in the inner ear, we examined *Vglut3* expression following AAV8-*Vglut3*-FLAG administration in *Vglut3*^{KO} mice. One microliter of AAV8-*Vglut3*-FLAG (titer: 2.04×10^{13} genome copies/mL) was injected into the inner ear, and cochlear whole mounts were prepared for immunofluorescence staining at 1, 2, 3, 5, and 14 days post-injection to assess gene expression (Figure 4A). In our previous study, we validated the feasibility of using a FLAG tag to label VGLUT3 protein.[27] Therefore, FLAG expression was used as a surrogate marker for VGLUT3 expression in this study. Immunofluorescence analysis revealed that the temporal and spatial expression pattern of FLAG in the cochlea closely resembled that of GFP observed in our previous experiments (Figures 4B, 4D, and 4F).

FLAG expression was first detected at 1 day post-injection, gradually increased over time, and peaked at 14 days (Figures 4B, 4D, and 4F). Similar to GFP, FLAG expression was observed throughout the apical, middle, and basal cochlear turns, with no significant regional delay in expression onset (Figures 4B, 4D, and 4F). These results suggest that the delivery and expression kinetics of *Vglut3* via AAV8 follow a pattern similar to that of GFP, further confirming the efficiency of AAV8-mediated gene transfer in the cochlea.

To quantify FLAG expression over time, we utilized ImageJ software to measure fluorescence intensity at various post-injection time points. To account for background fluorescence and ensure accurate quantification, the fluorescence intensity of IHCs was normalized by dividing it by the fluorescence intensity of the surrounding background area (Figures 4C, 4E, and 4G). The analysis confirmed a continuous increase in FLAG fluorescence intensity within IHCs following AAV8-*Vglut3*-FLAG injection. By day 1 post-injection, the fluorescence intensity was significantly elevated in the middle (1.16 ± 0.10 vs. 1.00 ± 0.08, n = 36 and 36, one-way ANOVA, $p < 0.05$) turn of the cochleae. By day 5 post-injection, FLAG expression in the apical (2.70 ± 1.11 vs. 1.00 ± 0.09, n = 38 and 36 IHCs, N = 3 mice per groups, one-way ANOVA, $p < 0.0001$), middle (2.33 ± 0.71 vs. 1.00 ± 0.08, n = 38 and 36 IHCs, N = 3 mice per groups, one-way ANOVA, $p < 0.0001$), and basal (2.11 ± 0.81 vs. 1.00 ± 0.04, n = 38 and 36 IHCs, N = 3 mice per groups, one-way ANOVA, $p < 0.0001$) turns of the cochlea exhibited a statistically significant increase compared to the control group. These findings indicate that AAV8-mediated transgene expression in the cochlea is initiated within the first few days post-injection, gradually intensifies, and achieves widespread distribution by one week, providing crucial insight into the kinetics of gene delivery for cochlear gene therapy. To address potential concerns about FLAG-induced structural interference or functional perturbation of VGLUT3, we performed additional validation experiments. The results demonstrated consistent expression patterns between FLAG-tagged and native VGLUT3, confirming that the FLAG tag did not significantly alter protein localization or compromise functional correlations (Figure S3).

To evaluate whether AAV8-*Vglut3*-FLAG injection induced any damage to cochlear hair cells, we quantified the survival rates of IHCs and OHCs at different post-injection time points across the apical, middle, and basal turns of the cochlea. Whole-mount cochlear preparations were immunostained with Myo7a at 1, 2, 3, 5, and 14 days post-injection to assess hair cell integrity. The results demonstrated that at all examined time points, both IHCs and OHCs remained intact, with no significant hair cell loss in any cochlear region compared to the control group (Figure S4). These findings indicate that AAV8-*Vglut3*-FLAG administration via PSCC does not compromise hair cell viability in the short term, further supporting the safety of this gene delivery approach for inner ear gene therapy.

Temporal changes in hearing thresholds of *Vglut3*^{KO} mice following gene therapy

To assess the temporal changes in auditory function following gene therapy, ABRs were recorded at multiple time points post-injection in *Vglut3*^{KO} mice (Figure 5). Baseline ABR thresholds were elevated in *Vglut3*^{KO} mice prior to AAV8-*Vglut3*-FLAG administration. By day 1 post-injection, ABR thresholds showed a significant reduction, indicating an early recovery of auditory function (Figure 5A). By day 1 post-injection, significant improvement in ABR thresholds was observed compared to pre-treatment baselines (83.8 ± 2.5, 80.0 ± 4.1, 73.8 ± 4.8, 73.8 ± 4.8, 78.8 ± 8.5 and 85.0 ± 4.1 dB vs. 90.0 ± 0 dB at 5.66, 8, 11.31, 16, 22.63, and 32 kHz; N = 4 and 10, two-way ANOVA, $p < 0.05$ at 11.31 and 16 kHz, Figure 5B). Strikingly, the most rapid recovery of ABR thresholds occurred within the first 24–48 h post-treatment, with treated *Vglut3*^{KO} mice exhibiting significantly improved thresholds by day 2 compared to day 1 post-injection. (65.0 ± 4.1, 58.8 ± 7.5, 45.0 ± 15.8, 50.0 ± 12.9, and 52.50 ± 17.6 dB vs. 83.8 ± 2.5, 80.0 ± 4.1, 73.8 ± 4.8, 73.8 ± 4.8, 78.8 ± 8.5, and 85.0 ± 4.1 dB at 5.66, 8, 11.31, 16, 22.63, and 32 kHz, $p < 0.05$. two-way ANOVA, N = 4 and 4, Figure 5B). By day 3 post-injection, ABR thresholds were significantly closer to WT levels (Figures 5B, 5C, and S5A–S5E). Hearing thresholds continued to improve over time, reaching a plateau by day 5, at which point they were fully restored to WT levels (Figures 5A and 5B), demonstrating the effectiveness of rescuing hearing function in *Vglut3*^{KO} mice.

Our longitudinal studies demonstrated that while transgene expression continued to increase over time, ABR thresholds remained stable at WT levels. This observation strongly suggests that partial rescue of IHCs is sufficient to restore baseline auditory function. We conducted additional experiments using a 50% reduced viral titer (1.02×10^{12} vg/mL) in *Vglut3*^{KO} mice. Remarkably, despite this significant reduction in viral titer, we observed complete restoration of ABR thresholds to WT levels within two weeks post-injection (Figure S5F). These compelling results provide direct experimental validation that the therapeutic threshold for functional rescue is indeed substantially lower than that required for maximal protein expression.

To better visualize the temporal changes in hearing thresholds, we plotted the ABR thresholds of each mouse at different time points following treatment. This analysis clearly illustrated the progressive improvement in hearing over time, with a notable reduction in the hearing threshold starting from day 1 post-injection (Figures 5D–5G). Additionally, we measured the ABR wave I amplitudes at different time points post-gene therapy. At 22, 16, and 11 kHz frequencies, a consistent increase in wave I amplitude was observed at a sound pressure level of 90 dB (Figure S6). This upward trend further supported the progressive recovery of auditory function in the treated mice, indicating an improvement in both the sensitivity and response of the cochlea to sound stimuli following gene therapy.

To evaluate the behavioral outcomes of gene therapy, we performed free-field sound detection tests by quantifying acoustic startle response rates to 90 dB stimuli in *Vglut3*^{WT}, untreated *Vglut3*^{KO}, and *Vglut3*^{KO} mice after two weeks of therapeutic intervention. The results demonstrated that all *Vglut3*^{WT} mice exhibited consistent

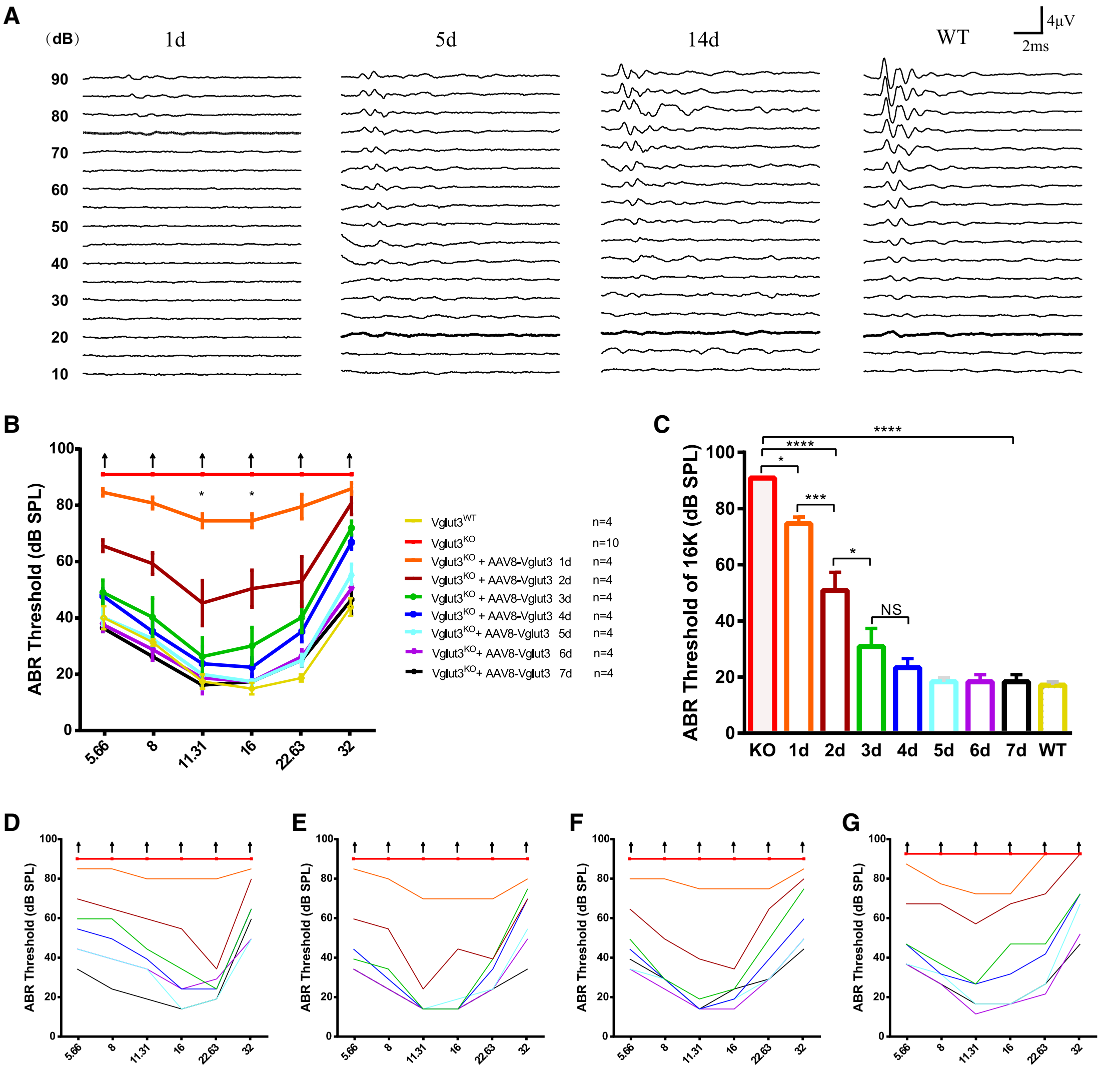

Figure 5. Temporal changes in hearing thresholds of *Vglut3^{KO}* mice following AAV8-*Vglut3*-FLAG gene therapy

(A) Representative ABR waveforms at 16 kHz recorded from *Vglut3^{KO}* mice at different time points post-injection, showing a progressive improvement in response amplitude and latency over time. (B) Quantification of ABR thresholds at multiple frequencies (5.66, 8, 11.31, 16, 22.63, and 32 kHz) in *Vglut3^{KO}* mice before and after AAV8-*Vglut3*-FLAG administration (*n* = 4–10 per group, two-way ANOVA, **p* < 0.05, ***p* < 0.01, and ****p* < 0.001). (C) Comparison of ABR thresholds at 16 kHz between treated *Vglut3^{KO}* mice and WT controls at different post-injection time points. (D–G) Individual ABR threshold tracking of each treated mouse at different post-injection time points, illustrating the temporal recovery of hearing function following gene therapy. Data are presented as mean ± SEM. Scale bars in (A): 2 ms (horizontal), 4 μV (vertical).

ear movement responses to acoustic stimulation, whereas untreated *Vglut3^{KO}* mice completely lacked this reflexive behavior. Notably, a full restoration of the acoustic startle response was observed in all gene therapy-treated *Vglut3^{KO}* mice at the 2-week post-treatment time point, indicating successful functional recovery of auditory reflex pathways (Figure S5G). These behavioral findings correlate with the therapeutic efficacy observed by ABR.

The functional relationship between hearing thresholds and FLAG fluorescence intensity

The dynamic changes in ABR thresholds following treatment were systematically analyzed in relation to the post-treatment time course. As illustrated in Figure 5, a significant downward trend in ABR thresholds was observed as the number of days post-treatment increased, indicating a progressive recovery of auditory function in the treated mice. To quantitatively assess this relationship, a regression analysis was performed using Excel to model the correlation between ABR thresholds and post-treatment days. The data were best fit by a logarithmic regression model, which revealed a strong negative correlation between the two variables (16kHz: $y = -35.96\ln(x) + 73.392$, $R^2 = 0.9881$, Figure 6A; 8kHz: $y = -30.78\ln(x) + 78.986$, $R^2 = 0.9717$, Figure S7A; 32kHz: $y = -18.33\ln(x) + 88.401$, $R^2 = 0.8957$, Figure S7C). These results collectively underscore a statistically significant and functionally relevant association between post-treatment

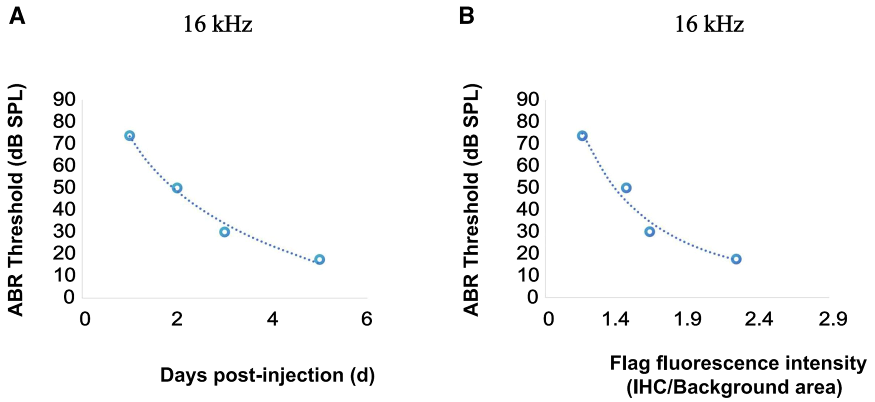

Figure 6. Quantitative analysis of ABR threshold recovery and its correlation with *Vglut3* expression
(A) Logarithmic regression curves depicting the time-dependent decline in ABR thresholds at 16 kHz following gene therapy. Individual data points (circles) show thresholds for each mouse (n = 4) at post-injection days 1–5. (B) Power-law regression curves illustrating the inverse relationship between FLAG fluorescence intensity (surrogate for *Vglut3* expression) and ABR thresholds at 16 kHz. Data points (circles) represent individual mice (n = 4), with fluorescence intensity quantified from cochlear sections and normalized to baseline.

duration and ABR threshold recovery, highlighting the efficacy of the intervention in restoring auditory sensitivity over time.

Based on our experimental results, we observed that following inner ear injection, the hearing thresholds (ABR) decreased as *Vglut3* expression progressively increased over time. To investigate the relationship between these two variables, we used Excel to calculate the functional correlation between the two datasets. As shown in Figure 6, the data demonstrate a clear inverse relationship between FLAG fluorescence intensity and hearing thresholds ($y = 76.296\ x^{-0.976}$ at 8 kHz frequency; $y = 104.29\ x^{-2.242}$ at 16 kHz frequency; $y = 98.888\ x^{-0.831}$ at 32 kHz frequency, Figures 6B, S6B, and S6D), with increasing *Vglut3* expression correlating with improved auditory function.

DISCUSSION

This study provides the first high-resolution timeline of gene expression kinetics and functional recovery following inner ear gene therapy in adult deaf mice. Using AAV8-mediated *Vglut3* delivery in *Vglut3*^{KO} mice, we demonstrate that transgene expression initiates within 24 h post-injection, with measurable hearing recovery detectable as early as day 1. ABR thresholds improved progressively, plateauing to WT levels by day 5, while FLAG-tagged VGLUT3 expression continued to increase through day 14. A nonlinear inverse correlation between fluorescence intensity and ABR thresholds during the first 5 days ($y = 104.29\ x^{-2.242}$ at 16 kHz) revealed a time-dependent therapeutic relationship, which dissolved upon threshold stabilization. Crucially, PSCC delivery of AAV8 proved safe, with no acute ototoxicity or hair cell loss. Our study demonstrates a significant advantage over conventional approaches by achieving rapid therapeutic efficacy in adult mice, in contrast to most gene therapy studies in hearing research that require several weeks to observe functional recovery due to their use of neonatal injection paradigms.

Our discovery of 24-h post-injection hearing recovery represents a radical departure from prior studies, which typically assessed outcomes at 1–2 weeks. In *Otof*$^{-/-}$ mice, ABR threshold improvements were first reported at 7 days post-treatment using dual AAV vectors,[13] while *Tmprss3*$^{A306T/A306T}$ models showed recovery at 4 weeks.[16] The rapid GFP and FLAG expression observed here—detectable in IHCs by day 1—aligns with recent work demonstrating AAV's superior transduction efficiency in the cochlea. However, the functional significance of this early expression was previously unrecognized. Our data reveal that even minimal VGLUT3 levels (e.g., 1.16-fold fluorescence increase in middle turn IHCs at day 1) suffice to initiate synaptic transmission, enabling partial hearing recovery (83.8 dB at 11.31 kHz vs. 90 dB baseline, $p < 0.05$). This suggests that therapeutic thresholds for functional rescue are far lower than those required for maximal protein expression, a critical insight for optimizing vector dosing.

While ABR thresholds normalized to WT levels by day 5 (Figure 5), FLAG fluorescence continued rising through day 14, achieving 6-fold increases in IHCs. Intriguingly, ABR wave I amplitudes—a measure of synaptic ribbon regeneration—kept increasing beyond day 5 (Figure S7), despite threshold stabilization. This temporal dissociation implies two distinct phases of recovery: (1) rapid threshold normalization driven by minimal VGLUT3 expression and (2) prolonged synaptic plasticity requiring sustained protein availability. The plateau in thresholds despite incomplete transgene coverage suggests redundancy in auditory circuitry, where partial rescue of IHCs suffices to restore baseline hearing. Such redundancy may explain why *Vglut3*^{KO} mice retain residual ABR responses despite complete loss of glutamate release, though further electrophysiological studies are needed to validate this hypothesis.

The inverse correlation between FLAG intensity and ABR thresholds during days 1–5 highlights the exquisite sensitivity of early hearing recovery to incremental increases in VGLUT3. The power-law relationship ($y = 104.29\ x^{-2.242}$ at 16 kHz) suggests that hearing improvement depends on the square of transgene expression levels during this phase. However, this relationship collapsed once thresholds stabilized (day 5), indicating a transition from dynamic repair to maintenance phases. Mechanistically, this may reflect saturation of vesicular glutamate transport capacity in IHCs, beyond which additional VGLUT3 provides diminishing returns.

The complete normalization of ABR thresholds at 5 days post-treatment contrasts with the attenuated wave I amplitudes,

a pattern mirroring the electrophysiological hallmark of hidden hearing loss (HHL) observed in human patients with cochlear synaptopathy.[29,30] The wave I deficit implicates persistent limitations in vesicular glutamate reloading dynamics, as *Vglut3* is critically required for packaging glutamate into synaptic vesicles at IHC ribbon synapses.[2,31] Our results further posit vesicular trafficking defects as a novel etiological axis in HHL pathogenesis, expanding beyond classical neurotrophin depletion hypotheses. Future studies should investigate combinatorial therapies targeting both vesicle loading (*Vglut3*) and synaptic stabilization (BDNF/neurotrophin-3) to achieve complete functional restoration.[32–35]

The absence of ABR threshold shifts (Figure 3) and 100% hair cell survival through day 14 validates the safety of AAV8 delivery via PSCC. This contrasts with earlier reports of transient threshold elevations following cochleostomy or round window injections, likely due to surgical trauma. Our minimally invasive approach—capitalizing on endolymphatic fluid circulation—achieved efficient cochlear transduction without disrupting vestibular function or IHC/OHC integrity. These results align with clinical trials using AAV delivery in humans, reinforcing the translational potential of this route.

While interesting, our findings are constrained to $Vglut3^{KO}$ mice and gene replacement strategies. Whether similar kinetics apply to gene editing (e.g., CRISPR-Cas9) or other deafness genes (e.g., *Tmc1* and *Otof*) remains untested. Human trials by Shu and Chai report hearing improvements unfolding over weeks—a slower trajectory than our murine data—potentially reflecting differences in cochlear size, AAV serotype tropism, or immune responses.[5,7,8] Additionally, our experimental model excludes hearing loss pathologies involving hair cell degeneration, where structural cochlear damage could compromise AAV-mediated therapeutic efficacy. This study's restriction to adult mice stems from immature auditory function in pre-hearing (P12–P14) animals,[36,37] precluding reliable post-treatment assessment during early postnatal development stages. The scarcity of adult-focused inner ear gene therapy studies may explain why this threshold-amplitude dissociation remains unreported. Nevertheless, the conserved inverse correlation between gene expression and functional outcomes suggests broader applicability of our kinetic framework.

By mapping the coordinated progression of gene expression and hearing recovery at unprecedented temporal resolution, this study revolutionizes our understanding of cochlear gene therapy. The discovery of day-1 therapeutic effects enables rapid screening of novel vectors and dosing regimens. The nonlinear time-response relationship provides a quantitative framework for optimizing onset of treatment efficacy, while the safety profile of PSCC delivery supports its clinical adoption. As gene therapy advances toward human trials, these insights will prove indispensable for bridging the gap between murine models and effective treatments for hereditary deafness.

MATERIALS AND METHODS

Animal models

All animals were housed in accredited barrier facilities under controlled environmental conditions, with standardized access to food and water. The study was approved by the Institutional Animal Care and Use Committee of the First Affiliated Hospital of Zhengzhou University (Henan Province, China). Male C57BL/6J mice (4–5 weeks old) were purchased from GemPharmatech Co., Ltd. (Nanjing, China), while $Vglut3^{KO}$ mice were obtained from the Institute of Neuroscience, CAS Center for Excellence in Brain Science and Intelligence Technology, Shanghai Institutes for Biological Sciences, Chinese Academy of Sciences.[38] All experiments were conducted in strict accordance with the 3Rs principles of animal research: reduction, replacement, and refinement. Mice of the same genotype were randomly assigned according to the experimental design. They were maintained on a 12-h light/dark cycle with ad libitum access to food and water.

Production of virus

AAVs were driven by a cytomegalovirus (CMV) promoter, obtained from ViGene Biosciences (Shandong, China). We constructed the pAAV-CMV-*Vglut3*-3×FLAG-P2A-GFP plasmid generated by inserting the *Vglut3* cDNA sequence into an AAV expression vector. The construct contains all critical regulatory elements including the CMV promoter, *Vglut3* coding sequence with C-terminal 3×FLAG tag, P2A self-cleaving peptide, and GFP reporter. The full vector schematic in Figure S5H clearly depicts the organization of all components between the AAV inverted terminal repeats (ITRs) (Figure S5H). The titers of AAV8-GFP and AAV8-*Vglut3*-FLAG were 3.87×10^{13} and 2.04×10^{13} genome copies/mL, respectively.

Microinjection into the inner ear of adult mice

Four-week-old mice underwent AAV delivery via surgical canalostomy. Prior to surgery, the mice were anesthetized. The right postauricular region was carefully shaved, and a 1 cm incision was made behind the ear to expose PSCC by separating the right pinna and sternocleidomastoid muscle.[39] A Bonn microprobe (Fine Science Tools, Foster City, CA) was used to perforate the PSCC, which was then connected to a Nanoliter 2000 micromanipulator (WPI, Sarasota, FL) via a glass micropipette (WPI) and a fine polyimide tube.[40] The tube tip was inserted into the PSCC, and the perforation site was sealed with tissue adhesive (3M Vetbond, St. Paul, MN) to facilitate AAV delivery.[39,40] The solution was injected at a controlled rate of 169 nL/min, with a total volume of 1 μL administered. Following injection, the tubing was carefully trimmed and sealed with tissue adhesive, and the incision was sutured using 7/0 sutures to ensure proper wound closure (Figure S1D).

Auditory testing

ABRs were recorded following anesthesia with an intraperitoneal injection of chloral hydrate (480 mg/kg). To prevent corneal desiccation, erythromycin ointment was applied to the eyes, and body temperature was maintained at approximately 37°C using a heating pad. Three subdermal needle electrodes were positioned at the vertex of the skull, the

mastoid region of the test ear, and the base of the tail. Prior to initiating ABR recordings, amplifier resistance was verified, and the soundproof chamber was securely sealed. Stimuli consisted of 3 ms short tone pips with 1 ms rise and fall cosine ramps, presented at a rate of 20 Hz via a computer-controlled acoustic stimulation system (Tucker-Davis Technologies). For each frequency and sound pressure level (SPL), 400 responses were recorded and averaged using BioSigRZ software (Tucker-Davis Technologies). All ABR measurements were performed by the same experimenter to ensure consistency. Hearing thresholds were determined as the lowest SPL that elicited a detectable ABR, measured in 5 dB decrements from 90 dB SPL across frequencies ranging from 5.66 to 32 kHz, as 90 dB SPL stimuli are commonly used in ABR measurements without inducing permanent threshold shifts.[30] The amplitude of ABR wave I was quantified as the peak-to-trough difference, serving as an indicator of neural response strength.

Sound detection in free field

To assess auditory function using sound detection in a free-field paradigm, the experiment should be conducted in a sound-attenuated chamber to minimize ambient noise interference. Mice are individually placed in testing cages and allowed 10–15 min of habituation to reduce stress responses before testing. Acoustic stimuli (16 kHz pure tone at 90 dB SPL, 1–2 s duration) are presented through a calibrated speaker system, with each trial carefully monitored for the presence or absence of ear movement responses (scored as 1 or 0, respectively). The response rate is subsequently calculated as the percentage of positive responses relative to the total number of mice, with this quantitative measure serving as an indicator of auditory sensitivity at the tested frequency.

$$\text{Response rate } (\%) = (\text{number of mice with ear movement responses} \; / \; \text{total number of mice}) \times 100\%$$

Immunohistochemistry

Cochleae were dissected from adult mice and perfused with 4% paraformaldehyde, followed by decalcification in 120 mM ethylenediaminetetraacetic acid (EDTA [pH 7.4]) for 3–4 h. The EDTA-decalcified cochleae were thoroughly rinsed with PBS (3×) before whole-mount preparation. Precise microdissection into apical, middle, and basal turns was performed using fine forceps under a stereomicroscope (Figures 1, 2, and 4). Tissue samples were blocked with 5% blocking buffer for one hour at room temperature and subsequently incubated overnight with primary antibodies, including anti-Myo7A (1:300; Proteus Biosciences) and anti-FLAG (1:200, AF519; Beyotime).

Following primary antibody incubation, tissues were washed three times with phosphate-buffered saline (PBS) and then incubated for one hour at room temperature with Alexa Fluor-conjugated secondary antibodies (1:500; Invitrogen) and Phalloidin (1:500, ab176759; Abcam). Confocal imaging was performed using a Zeiss LSM 880 laser scanning confocal microscope equipped with 20× and 63× glycerin-immersion objectives. Tonotopic cochlear maps were generated using the ImageJ plugin.

Statistical analysis

We use Excel to calculate the functional relationship between hearing and VGLUT3 expression. We enter paired X (normalized FLAG fluorescence ratio)-Y (ABR threshold) data, insert a scatterplot, add a trendline, select the best-fit function, and display the equation. Use LINEST() for regression coefficients and apply the equation in cells to compute values.

We utilized ImageJ software to quantify the grayscale value of FLAG expression in IHCs as a measure of fluorescence intensity. To minimize confounding factors, the values were normalized to the grayscale value of the background area.

Statistical analyses were conducted using Prism software (GraphPad, USA). One-way or two-way analysis of variance (ANOVA) was performed, followed by a Bonferroni post hoc test to determine statistical significance. Data are presented as the mean ± standard deviation (SD) in the text and as the mean ± standard error of the mean (SEM) in the figures. A significance threshold was set at $p < 0.05$. In the figures, statistical significance is denoted as follows: “NS” (not significant) for $p > 0.05$, * for $p < 0.05$, ** for $p < 0.01$, *** for $p < 0.001$, and **** for $p < 0.0001$.

DATA AVAILABILITY

The data are available from the corresponding author upon reasonable request.

ACKNOWLEDGMENTS

This work was supported by the China Postdoctoral Science Foundation (2024M752977), the Medical Science and Technology Research Plan Joint Construction Project of Henan Province (LHGJ20240279), and the Postdoctoral Research Foundation of the First Affiliated Hospital of Zhengzhou University (72137).

We also thank the Precision Medicine Center, Academy of Medical Sciences, Zhengzhou University, and the Key Laboratory for Research on Deafness Mechanism, Henan Provincial Health Commission (Henan Health Science and Education Letter 2021, no. 44) for their support.

AUTHOR CONTRIBUTIONS

X.Z. initiated and managed the project, while X.Z. and B.C. conceived and designed the experiments. T.Z. and M.L. performed most of the experiments and data analysis. All authors contributed to data analysis, interpretation, and presentation. X.Z. wrote the manuscript with contributions from all authors. The supplementary experiments to answer the reviewer’s questions were mainly completed by W.T. and R.Z.

DECLARATION OF INTERESTS

Neither the entire manuscript nor any part of its content has been published or accepted elsewhere, and this manuscript has not been submitted to any other journal. No portion

of the text has been copied from other published material. All authors have read and approved the final version of this manuscript.

SUPPLEMENTAL INFORMATION

Supplemental information can be found online at https://doi.org/10.1016/j.omtm.2025.101539.

REFERENCES

1. Xue, Y., Tao, Y., Wang, X., Wang, X., Shu, Y., Liu, Y., Kang, W., Chen, S., Cheng, Z., Yan, B., et al. (2023). RNA base editing therapy cures hearing loss induced by OTOF gene mutation. Mol. Ther. *31*, 3520–3530. https://doi.org/10.1016/j.ymthe.2023.10.019.

2. Seal, R.P., Akil, O., Yi, E., Weber, C.M., Grant, L., Yoo, J., Clause, A., Kandler, K., Noebels, J.L., Glowatzki, E., et al. (2008). Sensorineural deafness and seizures in mice lacking vesicular glutamate transporter 3. Neuron *57*, 263–275. https://doi.org/10.1016/j.neuron.2007.11.032.

3. Ruel, J., Emery, S., Nouvian, R., Bersot, T., Amilhon, B., Van Rybroek, J.M., Rebillard, G., Lenoir, M., Eybalin, M., Delprat, B., et al. (2008). Impairment of SLC17A8 encoding vesicular glutamate transporter-3, VGLUT3, underlies nonsyndromic deafness DFNA25 and inner hair cell dysfunction in null mice. Am. J. Hum. Genet. *83*, 278–292. https://doi.org/10.1016/j.ajhg.2008.07.008.

4. Akil, O., Seal, R.P., Burke, K., Wang, C., Alemi, A., During, M., Edwards, R.H., and Lustig, L.R. (2012). Restoration of hearing in the VGLUT3 knockout mouse using virally mediated gene therapy. Neuron *75*, 283–293. https://doi.org/10.1016/j.neuron.2012.05.019.

5. Lv, J., Wang, H., Cheng, X., Chen, Y., Wang, D., Zhang, L., Cao, Q., Tang, H., Hu, S., Gao, K., et al. (2024). AAV1-hOTOF gene therapy for autosomal recessive deafness 9: a single-arm trial. Lancet (London, England) *403*, 2317–2325. https://doi.org/10.1016/s0140-6736(23)02874-x.

6. Gao, X., Tao, Y., Lamas, V., Huang, M., Yeh, W.H., Pan, B., Hu, Y.J., Hu, J.H., Thompson, D.B., Shu, Y., et al. (2018). Treatment of autosomal dominant hearing loss by *in vivo* delivery of genome editing agents. Nature *553*, 217–221. https://doi.org/10.1038/nature25164.

7. Qi, J., Xu, L., Zeng, F.G., and Chai, R. (2025). OTOF-related gene therapy: a new way but a long road ahead. Lancet (London, England) *405*, 777–779. https://doi.org/10.1016/s0140-6736(25)00248-x.

8. Wang, H., Chen, Y., Lv, J., Cheng, X., Cao, Q., Wang, D., Zhang, L., Zhu, B., Shen, M., Xu, C., et al. (2024). Bilateral gene therapy in children with autosomal recessive deafness 9: single-arm trial results. Nat. Med. *30*, 1898–1904. https://doi.org/10.1038/s41591-024-03023-5.

9. Wei, G., Qiu, S., Gao, X., Zheng, L., Chen, Y., Ma, Y., Feng, H., Yang, J., Dong, G., Nie, H., et al. (2025). Single administration of AAV-mAtp6v1b2 gene therapy rescues hearing and vestibular disorders caused by Atp6v1b2-induced lysosomal dysfunction in hair cells. Adv. Sci. e2408878. https://doi.org/10.1002/advs.202408878.

10. Moser, T., Chen, H., Kusch, K., Behr, R., and Vona, B. (2024). Gene therapy for deafness: are we there now? EMBO Mol. Med. *16*, 675–677. https://doi.org/10.1038/s44321-024-00058-6.

11. Pan, B., Askew, C., Galvin, A., Heman-Ackah, S., Asai, Y., Indzhykulian, A.A., Jodelka, F.M., Hastings, M.L., Lentz, J.J., Vandenberghe, L.H., et al. (2017). Gene therapy restores auditory and vestibular function in a mouse model of Usher syndrome type 1c. Nat. Biotechnol. *35*, 264–272. https://doi.org/10.1038/nbt.3801.

12. Qi, J., Tan, F., Zhang, L., Zhou, Y., Zhang, Z., Sun, Q., Li, N., Fang, Y., Chen, X., Wu, Y., et al. (2024). Critical role of TPRN rings in the stereocilia for hearing. Mol. Ther. *32*, 204–217. https://doi.org/10.1016/j.ymthe.2023.11.011.

13. Akil, O., Dyka, F., Calvet, C., Emptoz, A., Lahlou, G., Nouaille, S., Boutet de Monvel, J., Hardelin, J.P., Hauswirth, W.W., Avan, P., et al. (2019). Dual AAV-mediated gene therapy restores hearing in a DFNB9 mouse model. Proc. Natl. Acad. Sci. USA *116*, 4496–4501. https://doi.org/10.1073/pnas.1817537116.

14. Al-Moyed, H., Cepeda, A.P., Jung, S., Moser, T., Kügler, S., and Reisinger, E. (2019). A dual-AAV approach restores fast exocytosis and partially rescues auditory function in deaf otoferlin knock-out mice. EMBO Mol. Med. *11*, e9396. https://doi.org/10.15252/emmm.201809396.

15. Yoshimura, H., Shibata, S.B., Ranum, P.T., Moteki, H., and Smith, R.J.H. (2019). Targeted Allele Suppression Prevents Progressive Hearing Loss in the Mature Murine Model of Human TMC1 Deafness. Mol. Ther. *27*, 681–690. https://doi.org/10.1016/j.ymthe.2018.12.014.

16. Du, W., Ergin, V., Loeb, C., Huang, M., Silver, S., Armstrong, A.M., Huang, Z., Gurumurthy, C.B., Staecker, H., Liu, X., and Chen, Z.Y. (2023). Rescue of auditory function by a single administration of AAV-TMPRSS3 gene therapy in aged mice of human recessive deafness DFNB8. Mol. Ther. *31*, 2796–2810. https://doi.org/10.1016/j.ymthe.2023.05.005.

17. Emptoz, A., Michel, V., Lelli, A., Akil, O., Boutet de Monvel, J., Lahlou, G., Meyer, A., Dupont, T., Nouaille, S., Ey, E., et al. (2017). Local gene therapy durably restores vestibular function in a mouse model of Usher syndrome type 1G. Proc. Natl. Acad. Sci. USA *114*, 9695–9700. https://doi.org/10.1073/pnas.1708894114.

18. Tan, F., Chu, C., Qi, J., Li, W., You, D., Li, K., Chen, X., Zhao, W., Cheng, C., Liu, X., et al. (2019). AAV-ie enables safe and efficient gene transfer to inner ear cells. Nat. Commun. *10*, 3733. https://doi.org/10.1038/s41467-019-11687-8.

19. Tao, Y., Liu, X., Yang, L., Chu, C., Tan, F., Yu, Z., Ke, J., Li, X., Zheng, X., Zhao, X., et al. (2022). AAV-ie-K558R mediated cochlear gene therapy and hair cell regeneration. Signal Transduct. Target. Ther. *7*, 109. https://doi.org/10.1038/s41392-022-00938-8.

20. Tao, Y., Huang, M., Shu, Y., Ruprecht, A., Wang, H., Tang, Y., Vandenberghe, L.H., Wang, Q., Gao, G., Kong, W.J., and Chen, Z.Y. (2018). Delivery of Adeno-Associated Virus Vectors in Adult Mammalian Inner-Ear Cell Subtypes Without Auditory Dysfunction. Hum. Gene Ther. *29*, 492–506. https://doi.org/10.1089/hum.2017.120.

21. Kang, W., Zhao, X., Sun, Z., Dong, T., Jin, C., Tong, L., Zhu, W., Tao, Y., and Wu, H. (2020). Adeno-associated virus vector enables safe and efficient Cas9 activation in neonatal and adult Cas9 knockin murine cochleae. Gene Ther. *27*, 392–405. https://doi.org/10.1038/s41434-020-0124-1.

22. Isgrig, K., McDougald, D.S., Zhu, J., Wang, H.J., Bennett, J., and Chien, W.W. (2019). AAV2.7m8 is a powerful viral vector for inner ear gene therapy. Nat. Commun. *10*, 427. https://doi.org/10.1038/s41467-018-08243-1.

23. Xue, Y., Hu, X., Wang, D., Li, D., Li, Y., Wang, F., Huang, M., Gu, X., Xu, Z., Zhou, J., et al. (2022). Gene editing in a Myo6 semi-dominant mouse model rescues auditory function. Mol. Ther. *30*, 105–118. https://doi.org/10.1016/j.ymthe.2021.06.015.

24. Zhao, X., Jin, C., Dong, T., Sun, Z., Zheng, X., Feng, B., Cheng, Z., Li, X., Tao, Y., and Wu, H. (2020). Characterization of promoters for adeno-associated virus mediated efficient Cas9 activation in adult Cas9 knock-in murine cochleae. Hear. Res. *394*, 107999. https://doi.org/10.1016/j.heares.2020.107999.

25. Petit, C., Bonnet, C., and Safieddine, S. (2023). Deafness: from genetic architecture to gene therapy. Nat. Rev. Genet. *24*, 665–686. https://doi.org/10.1038/s41576-023-00597-7.

26. Jiang, L., Wang, D., He, Y., and Shu, Y. (2023). Advances in gene therapy hold promise for treating hereditary hearing loss. Mol. Ther. *31*, 934–950. https://doi.org/10.1016/j.ymthe.2023.02.001.

27. Zhao, X., Liu, H., Liu, H., Cai, R., and Wu, H. (2022). Gene Therapy Restores Auditory Functions in an Adult Vglut3 Knockout Mouse Model. Hum. Gene Ther. *33*, 729–739. https://doi.org/10.1089/hum.2022.062.

28. Ono, K., Keller, J., López Ramírez, O., González Garrido, A., Zobeiri, O.A., Chang, H.H.V., Vijayakumar, S., Ayiotis, A., Duester, G., Della Santina, C.C., et al. (2020). Retinoic acid degradation shapes zonal development of vestibular organs and sensitivity to transient linear accelerations. Nat. Commun. *11*, 63. https://doi.org/10.1038/s41467-019-13710-4.

29. Bakay, W.M.H., Anderson, L.A., Garcia-Lazaro, J.A., McAlpine, D., and Schaette, R. (2018). Hidden hearing loss selectively impairs neural adaptation to loud sound environments. Nat. Commun. *9*, 4298. https://doi.org/10.1038/s41467-018-06777-y.

30. Liu, H., Liu, H., Wang, L., Song, L., Jiang, G., Lu, Q., Yang, T., Peng, H., Cai, R., Zhao, X., et al. (2023). Cochlear transcript diversity and its role in auditory functions implied by an otoferlin short isoform. Nat. Commun. *14*, 3085. https://doi.org/10.1038/s41467-023-38621-3.

31. Obholzer, N., Wolfson, S., Trapani, J.G., Mo, W., Nechiporuk, A., Busch-Nentwich, E., Seiler, C., Sidi, S., Söllner, C., Duncan, R.N., et al. (2008). Vesicular glutamate transporter 3 is required for synaptic transmission in zebrafish hair cells. J. Neurosci. *28*, 2110–2118. https://doi.org/10.1523/jneurosci.5230-07.2008.

32. Gunewardene, N., Lam, P., Ma, Y., Caruso, F., Wagstaff, S., Richardson, R.T., and Wise, A.K. (2022). Pharmacokinetics and biodistribution of supraparticle-delivered neurotrophin 3 in the guinea pig cochlea. J. Control. Release *342*, 295–307. https://doi.org/10.1016/j.jconrel.2021.12.037.

33. Ji, L., Borges, B.C., Martel, D.T., Wu, C., Liberman, M.C., Shore, S.E., and Corfas, G. (2024). From hidden hearing loss to supranormal auditory processing by neurotrophin 3-mediated modulation of inner hair cell synapse density. PLoS Biol. *22*, e3002665. https://doi.org/10.1371/journal.pbio.3002665.

34. Mukherjee, S., Kuroiwa, M., Oakden, W., Paul, B.T., Noman, A., Chen, J., Lin, V., Dimitrijevic, A., Stanisz, G., and Le, T.N. (2022). Local magnetic delivery of adeno-associated virus AAV2(quad Y-F)-mediated BDNF gene therapy restores hearing after noise injury. Mol. Ther. *30*, 519–533. https://doi.org/10.1016/j.ymthe.2021.07.013.

35. Min, X., Deng, X.H., Lao, H., Wu, Z.C., Chen, Y., Luo, Y., Wu, H., Wang, J., Fu, Q.L., and Xiong, H. (2023). BDNF-enriched small extracellular vesicles protect against noise-induced hearing loss in mice. J. Control. Release *364*, 546–561. https://doi.org/10.1016/j.jconrel.2023.11.007.

36. Sun, Y., Ren, M., Zhang, Y., Li, S., Luo, Z., Sun, S., He, S., Wang, G., Zhang, D., Mansour, S.L., et al. (2025). Casz1 is required for both inner hair cell fate stabilization and outer hair cell survival. Science (New York, N.Y.) *388*, eado4930. https://doi.org/10.1126/science.ado4930.

37. Bi, Z., Li, X., Ren, M., Gu, Y., Zhu, T., Li, S., Wang, G., Sun, S., Sun, Y., and Liu, Z. (2022). Development and transdifferentiation into inner hair cells require Tbx2. Natl. Sci. Rev. *9*, nwac156. https://doi.org/10.1093/nsr/nwac156.

38. Zhang, H., Pan, H., Zhou, C., Wei, Y., Ying, W., Li, S., Wang, G., Li, C., Ren, Y., Li, G., et al. (2018). Simultaneous zygotic inactivation of multiple genes in mouse through CRISPR/Cas9-mediated base editing. Development (Cambridge, England) *145*, dev168906. https://doi.org/10.1242/dev.168906.

39. Kim, J., Maldonado, J., Pan, D.W., Quiñones, P.M., Zenteno, S., Oghalai, J.S., and Ricci, A.J. (2024). Semicircular canal drug delivery safely targets the inner ear perilymphatic space. JCI insight *9*, e173052. https://doi.org/10.1172/jci.insight.173052.

40. Zhao, Y., Zhang, L., Wang, D., Chen, B., and Shu, Y. (2022). Approaches and Vectors for Efficient Cochlear Gene Transfer in Adult Mouse Models. Biomolecules *13*, 38. https://doi.org/10.3390/biom13010038.

OMTM, Volume *33*

Supplemental information

Rapid cochlear gene therapy in adult deaf mice: *Vglut3* rescue via AAV8 achieves day-1 hearing restoration

Ting Zhang, Rongqun Zhai, Mengli Liu, Hongen Xu, Liang Wang, Wenxue Tang, Bei Chen, and Xingle Zhao

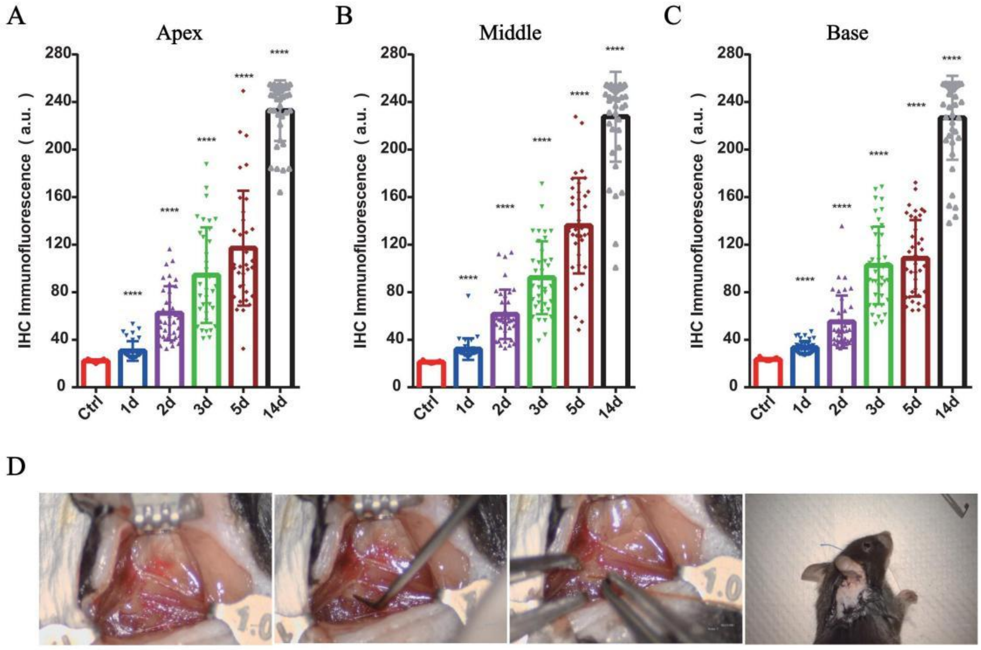

Figure S1. Quantification of GFP Fluorescence Intensity in the Cochlea Following AAV8-GFP Injection.

(A–C) Quantification of GFP fluorescence intensity in the apical (A), middle (B), and basal (C) turns of the cochlea at different time points post-injection (1, 2, 3, 5, and 14 days). Data are presented as mean ± SD. ****$p < 0.0001$. (D) Images of critical step of the adult posterior semicircular canal (PSCC) surgical approach.

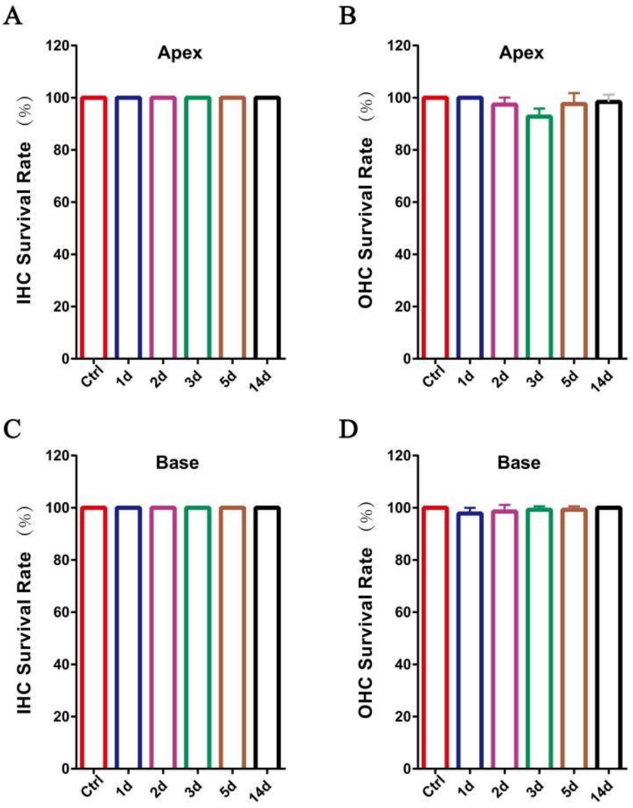

Figure S2. Hair Cell Survival in the Apical and Basal Turns of the Cochlea Following AAV8-GFP Injection

(A-D) Quantification of IHC and OHC survival rates in the apical (A, B) and basal (C, D) turns at various post-injection time points.Data are presented as mean ± SEM (N = 3, one-way ANOVA, $p > 0.05$)..

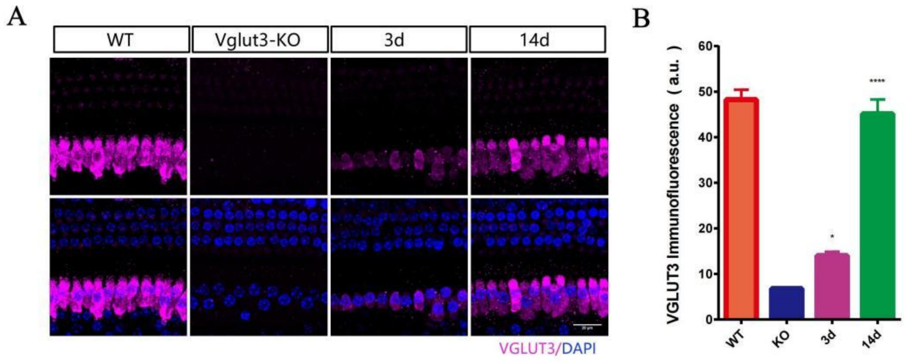

Figure S3. Temporal Dynamics of VGLUT3 Expression in the Adult Mouse Cochlea Following AAV8-*Vglut3*-FLAG Gene Therapy.

(A) Representative immunofluorescence images showing VGLUT3 expression in the middle turn of the cochlea at 3 and 14 days post-injection. Scale bar: 20 µm. (B) Quantification of VGLUT3 fluorescence intensity in the middle cochlear turn at different post-injection time points. Data are presented as mean ± SEM (n = 34–36 IHCs, N=3 mice per group). One-way ANOVA, *p < 0.05, ****p < 0.0001.

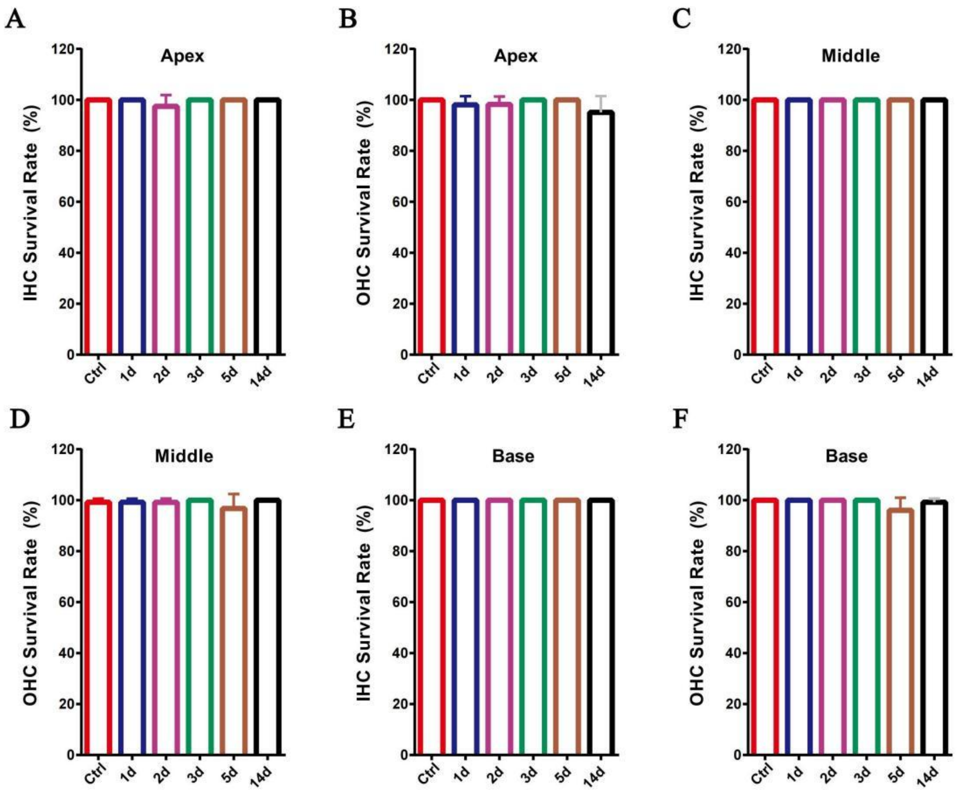

Figure S4. Hair cell survival in the apical, middle, and basal turns of the cochlea following AAV8-*Vglut3*-FLAG injection.

(A-F) Quantification of IHC and OHC survival rates in the apical (A, B), middle (C, D), and basal (E, F) cochlear turns at 1, 2, 3, 5, and 14 days post-injection. Data are presented as mean ± SEM (N = 3, one-way ANOVA, p > 0.05)..

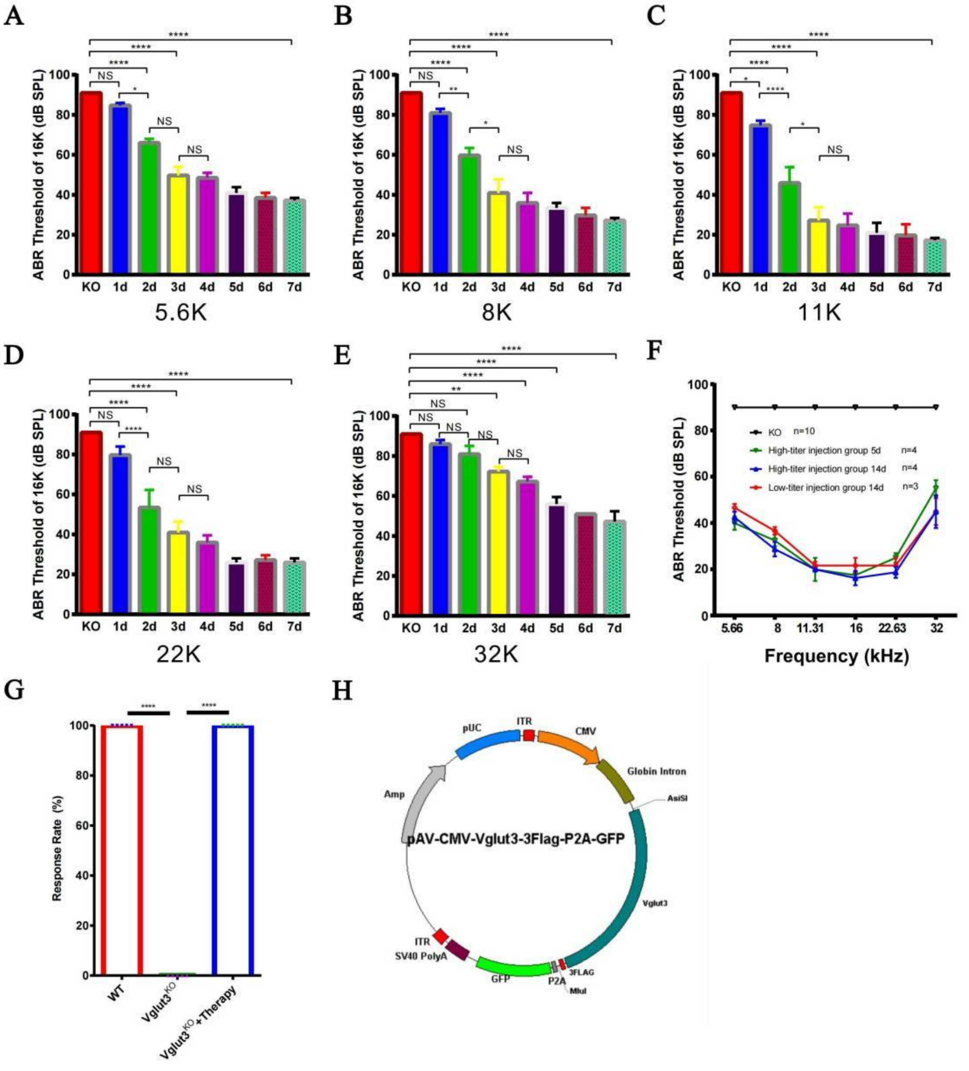

Figure S5. ABR thresholds Changes Following AAV8-*Vglut3*-FLAG Gene Therapy.

(A-E) Comparison of ABR thresholds at 5.66(A), 8(B), 11.31(C), 22.63(D), 32 (E)kHz between untreated $Vglut3^{KO}$ mice and treated $Vglut3^{KO}$ at different post-injection time points. (F) Quantification of ABR thresholds in $Vglut3^{KO}$ Mice with different Viral Titers. (G) Ear movement response rates to 90 dB stimuli in WT, $Vglut3^{KO}$, and $Vglut3^{KO}$ mice after 2 weeks of treatment. (H) Schematic representation of the vector.

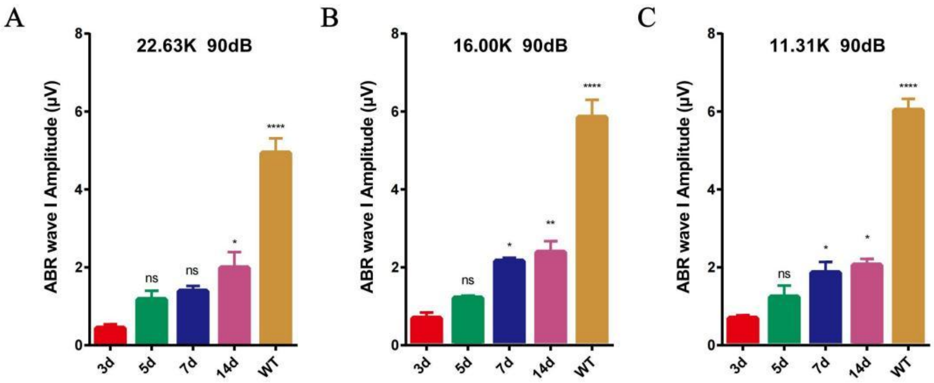

Figure S6. ABR Wave I Amplitude Changes Following AAV8-*Vglut3*-FLAG Gene Therapy.

(A-C) Quantification of ABR wave I amplitudes at 11.31 kHz (A), 16 kHz (B), and 22.63 kHz (C) at a sound pressure level of 90 dB across different post-injection time points. Data are presented as mean ± SEM.

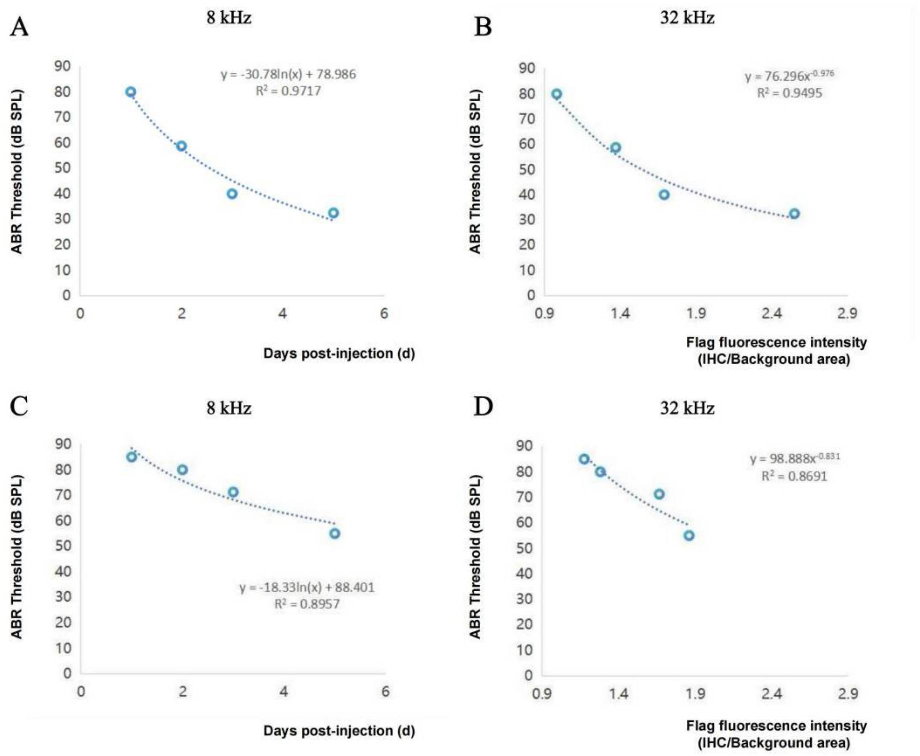

Figure S7. Quantitative Analysis of ABR Threshold Recovery and Its Correlation with *Vglut3* Expression.

(A) Logarithmic regression curves depicting the time-dependent decline in ABR thresholds at 8 kHz following gene therapy. (B) Power-law regression curves

illustrating the inverse relationship between FLAG fluorescence intensity (surrogate for *Vglut3* expression) and ABR thresholds at 8 kHz. (C) Logarithmic regression curves depicting the time-dependent decline in ABR thresholds at 32 kHz following gene therapy. (D) Power-law regression curves illustrating the inverse relationship between FLAG fluorescence intensity (surrogate for *Vglut3* expression) and ABR thresholds at 32 kHz.
